# Exploring engineering strategies that enhance de novo production of exotic cyclopropane fatty acids in *Saccharomyces cerevisiae*

**DOI:** 10.1002/biot.202300694

**Published:** 2024-02-25

**Authors:** Wei Jiang, Huadong Peng, Lizhong He, Rodrigo Lesma‐Amaro, Victoria S. Haritos

**Affiliations:** ^1^ Department of Chemical and Biological Engineering Monash University Clayton Victoria Australia; ^2^ Imperial College Centre for Synthetic Biology Imperial College London London UK; ^3^ Department of Bioengineering Imperial College London London UK

**Keywords:** exotic fatty acid, lipid metabolism, metabolic engineering, synthetic biology, triacylglycerol

## Abstract

Cycloalkanes have broad applications as specialty fuels, lubricants, and pharmaceuticals but are not currently available from renewable sources, whereas, production of microbial cycloalkanes such as cyclopropane fatty acids (CFA) has bottlenecks. Here, a systematic investigation was undertaken into the biosynthesis of CFA in *Saccharomyces cerevisiae* heterologously expressing bacterial CFA synthase. The enzyme catalyzes formation of a 3‐membered ring in unsaturated fatty acids. Monounsaturated fatty acids in phospholipids (PL) are the site of CFA synthesis; precursor *cis‐*Δ9 C16 and C18 fatty acids were enhanced through *OLE1* and *SAM2 over*expression which enhanced CFA in PL. CFA turnover from PL to storage in triacylglycerols (TAG) was achieved by phospholipase *PBL2* overexpression and acyl‐CoA synthase to increase flux to TAG. Consequently, CFA storage as TAG reached 12 mg g^−1^ DCW, improved 3‐fold over the base strain and >22% of TAG was CFA. Our research improves understanding of cycloalkane biosynthesis in yeast and offers insights into processing of other exotic fatty acids.

## Graphical Abstract and Lay Summary

This study presents a comprehensive exploration of the synthesis mechanism of cyclopropane fatty acid (CFA) in the yeast *S. cerevisiae*. It offers an in‐depth analysis of the synthesis site, identifies key production bottlenecks, and delves into the intricacies of CFA transfer among different lipid species.

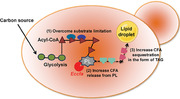

## 1 INTRODUCTION

Cycloalkane chemicals find broad and significant industrial applications in fields such as lubrication and pharmaceuticals industries, owing to their unique characteristics. The closed‐ring structure of cycloalkanes imparts them with excellent lubricating rigidity and stability.^[^ ^1^ ^]^ Cyclopropane fatty acids (CFA) are a specific kind of cycloalkane that have a three‐carbon ring attached to a long hydrocarbon chain. Currently, cycloalkanes are predominantly obtained through crude oil or natural gas refining processes.^[^ ^2^ ^]^ However, biosynthesis offers a promising alternative to traditional production methods^[^ ^3^ ^]^ presenting sustainable, environmentally friendly, and cost‐effective advantages.^[^ ^3^, ^4^ ^]^ Significant progress has been made in their biosynthesis. Engineered iterative polyketide synthases (iPKSs) expressed in *Streptomyces coelicolor* successfully produced polycyclopropanated fatty acids.^[^ ^5^ ^]^ While most bacterial platform demonstrated the production of highly diversified cycloalkane fatty acid moieties, they were not stored as triacylglycerols (TAG) in this study. TAG has the advantage of being a stable and convenient mechanism to accumulate significant quantities of exotic lipids.

For several decades, it has been known that CFA are present in the phospholipids (PL) of numerous bacterial species such as *E. coli*, *Rhodobacter sphaeroides*, etc.^[^ ^6^ ^]^ CFA are synthesized through the addition of a methylene group, which originates from the methyl group of S‐adenosylmethionine (SAM), to the carbon‐carbon double bond of unsaturated fatty acids^[^ ^6^ ^]^ Increasing the availability of substrates such as SAM and unsaturated fatty acids can potentially enhance CFA production. One strategy involves the expression of S‐adenosylmethionine synthase 2 (*ScSAM2*), which facilitates the synthesis of AdoMet through the reaction between methionine and ATP.^[^ ^7^ ^]^ Additionally, unsaturated fatty acid content of PL can be increased by overexpressing Δ9 desaturase, an enzyme that catalyzes the formation of a *cis‐*double bond between carbons 9 and 10 of palmitoyl (16:0) and stearoyl (18:0) CoA to form C16:1 and C18:1,^[^ ^8^ ^]^ respectively.

Prior research employed *Escherichia coli* synthase for CFA production and storage in seed oils of plants including *Arabidopsis thaliana* and *Camelina sativa*,^[^ ^9^ ^]^ however, CFA accumulation led to reduced seed germination and establishment, hampering growth of new seedlings.^[^ ^10^, ^11^ ^]^ Earlier studies demonstrated that expression of bacterial CFA synthase genes in yeasts such as *S. cerevisiae* and the oleaginous yeast *Yarrowia lipolytica*, resulted in accumulation of CFA.^[^ ^12^, ^13^, ^14^, ^15^ ^]^ General lipid pathway engineering in *S. cerevisiae*,^[^ ^14^ ^]^ CFA synthase gene candidate screening,^[^ ^12^ ^]^ promoter engineering^[^ ^15^ ^]^ and bioprocess engineering^[^ ^13^, ^15^ ^]^ in *Y.  lipolytica* strains resulted in improved yields of CFA up to 3.06 g L^−1^.^[^ ^15^ ^]^ However, no systematic research into the site of synthesis and potential bottlenecks in production and transfer of CFA between lipid species has been conducted to date.

The yeast *S. cerevisiae* is a reliable model organism to study complex fatty acid metabolism due to its safety, ease of handling and maturity of genome editing tools.^[^ ^16^ ^]^ Besides, precision fermentation of nutritional, industrial, and food lipids using yeasts is a highly promising and rapidly advancing research field, driven by the recognition that this alternative production approach holds the potential for cost‐effectiveness and sustainability surpassing traditional chemical engineering methods.

**FIGURE 1:**
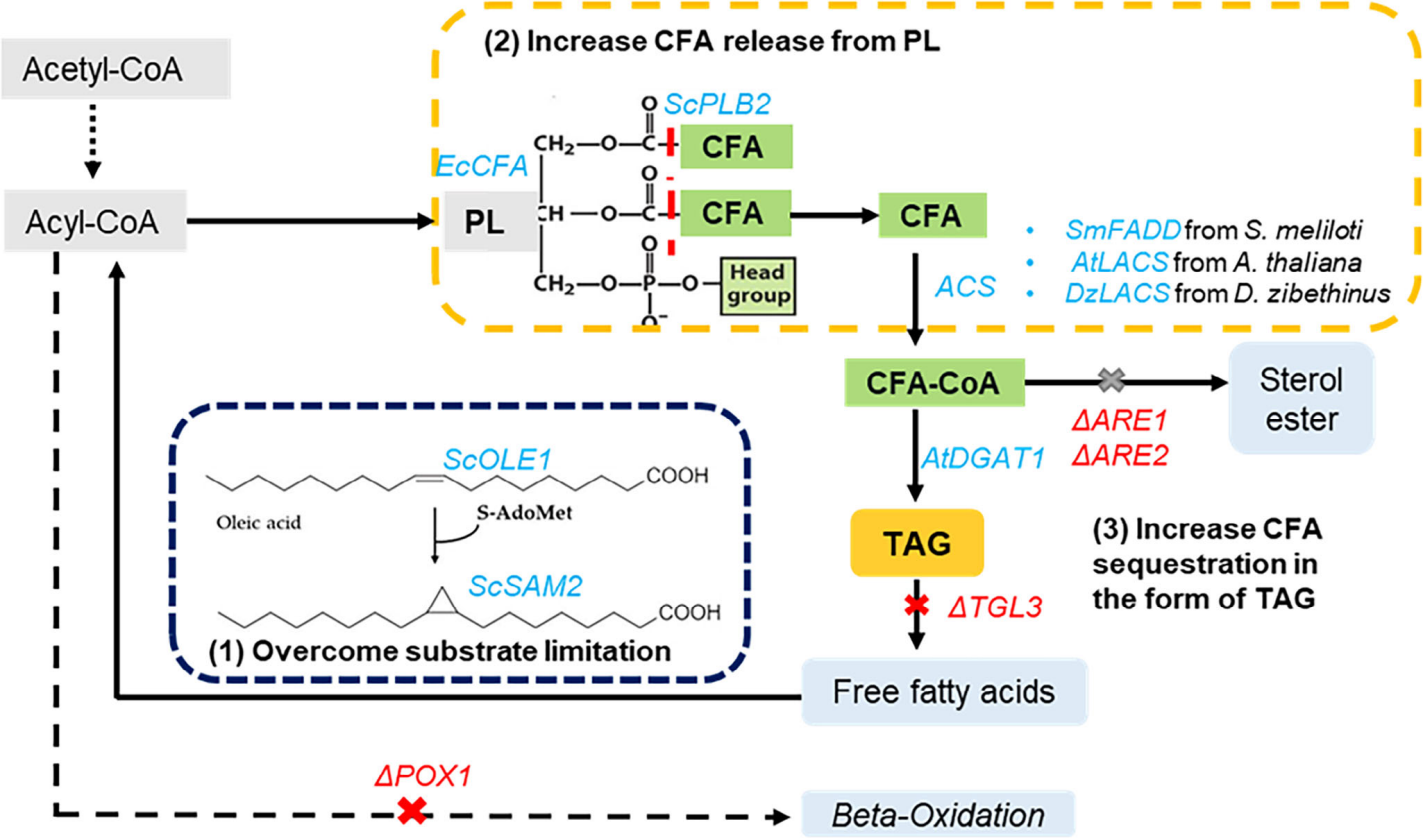
Summary of the pathways investigated for their impact on CFA content in triacylglycerol in *S. cerevisiae*, which encompassed three strategies (1) overcoming substrate limitation of *E. coli CFA* synthase by overexpressing *ScOLE1* and *ScSAM2*, (2) increasing CFA release from PL by overexpressing *ScPLB2* and converting CFA free fatty acids to CFAs‐CoA by expressing acyl‐CoA enzymes and inhibiting β‐oxidation of fatty acids by deleting *POX1* (3) enhancing CFA storage in TAG via increasing TAG accumulation by expression of *AtDGAT1*, preventing TAG degradation by deleting *TGL3*. The genes recombinantly expressed or overexpressed in *S. cerevisiae* are shown in blue text, genes deleted are red. *ScOLE1*: Δ9 fatty acid desaturase, *ScSAM2*: S‐adenosylmethionine synthase, *AtDAGT1*: diacylglycerol acyltransferase from *Arabidopsis thaliana*, *SmFADD*: fatty acyl‐CoA synthetase from *Sinorhizobium meliloti, AtLACS*: long‐chain acyl‐CoA synthase from *A. thaliana*, *DuLACS*: long‐chain acyl‐CoA synthase from *Durio zibethinus*, *ΔTGL3*: the deletion of triglyceride lipase 3, *ΔPOX1*: the deletion of fatty‐acyl coenzyme A oxidase, *ΔARE1&ΔARE2*: the deletion of acyl‐CoA sterol acyltransferase.

In this study, we aim to gain a comprehensive understanding of the synthesis mechanism of CFA in the yeast *S. cerevisiae*, including identifying the synthesis site, uncovering production bottlenecks, and exploring the transfer of CFA among various lipid species. We began by identifying the subcellular localization of the *EcCFA* synthase enzyme and investigated how native yeast lipid handling enzymes respond to the exotic fatty acid CFA. Additionally, we conducted a detailed analysis of CFA positioning within PL. To further our understanding, we proposed metabolic engineering strategies aimed at enhancing various facets of CFA metabolism. These strategies involved addressing substrate limitation of *EcCFA* synthase, increasing CFA release from PL and enhancing CFA storage in TAG (Figure 1). By employing this approach, our study provides fundamental insights into the biosynthesis of exotic fatty acids in yeast, paving the way for potential applications and further research in this field.

## 2 RESULTS AND DISCUSSION

### 2.1 *EcCFA* synthase localizes to both the plasma and internal membranes when heterologously expressed in *S. cerevisiae*

The enzyme CFA synthase associates with the lipid bilayers of the plasma membrane of *E. coli* and catalyzes the methylenation of unsaturated moieties of PL in the lipid bilayer of the bacterium.^[^ ^17^, ^18^ ^]^ However, there are no prior reports that describe the localization of heterologously expressed *EcCFA* synthase in eukaryotic cells which natively lack the gene. In this study, the subcellular localization of expressed *EcCFA* synthase was investigated via confocal microscopy of cells expressing the enzyme fused to green fluorescent protein (GFP) at the N‐terminus of the enzyme, to determine its main site of action.

The expressed *EcCFA‐GFP* fusion protein maintained its cyclopropanation function as demonstrated by the conversion of yeast fatty acids, which produced 11.68 ± 0.39% CFA of total fatty acids in the cell (Figure S1A). Fluorescence from *EcCFA‐GFP* was observed on the plasma membrane as shown in Figure S1B1,C1 and the green fluorescent signal was also visualized in other locations in recombinant yeast cells. To explore whether *EcCFA‐GFP* was also localized to the membranes of mitochondria, the yeast strain was stained with a mitochondria‐specific stain and visualized by confocal microscopy as shown in Figure S1B2. The partially or entirely overlapped fluorescent signal generated by *EcCFA‐GFP* and the mitochondrial dye indicated that cyclopropane synthase was also associated with the mitochondrial membrane (Figure S1B3,B4). Furthermore, the *EcCFA‐GFP* protein was also found to be associated with the lipid droplets; BODIPY 558/568 C12 neutral lipid dye was used to stain lipid droplets in the yeast strain as shown by the red fluorescence in Figure S1C2. The overlapped fluorescence of *EcCFA‐GFP* and the lipid stain suggested the cyclopropane synthase also localized to the membrane of lipid droplets in Figure S1C3,C4. Based on the findings from our investigation, it can be deduced that the heterologously expressed *EcCFA* synthase in *S. cerevisiae* localizes to both plasma and internal membranes. These results offer valuable insights into the subcellular distribution of *EcCFA* synthase within the yeast host and sites of catalysis, aiding in the identification of potential bottlenecks and the optimization of metabolic engineering strategies.

### 2.2 Native yeast lipid handling enzymes can metabolize CFA supplied exogenously

**FIGURE 2:**
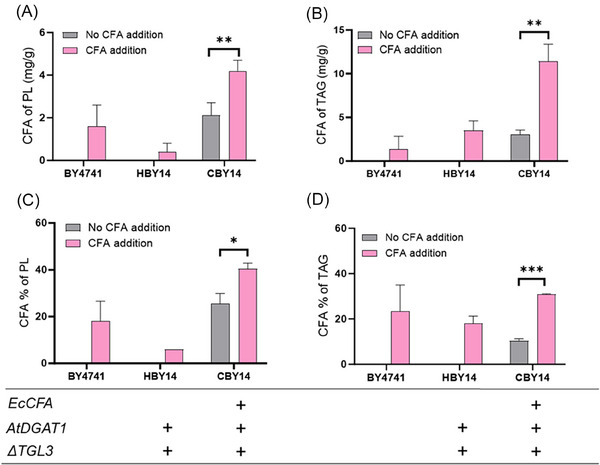
CFA content in phospholipid and triacylglycerol fractions of wild type BY4741, HBY14 and CBY14 strains supplemented with (pink bars) and without CFA (gray bars) in media. (A) CFA content of PL (mg g^−1^), (B) CFA content of TAG (mg g^−1^), (C) percentage of CFA in PL fraction (%), (D) percentage of CFA in TAG fraction (%). “+”, genes expression. Values are means of triplicate experiments, error bars means standard deviation. “*”, “**”, “***” denotes the effect of the factor was significant at *p* ≤ 0.05, *p* ≤ 0.01, and *p* ≤ 0.001, respectively. A two tailed Student's t‐test was used with BY4741 as the control strain.

*S. cerevisiae* import fatty acids supplied exogenously in the media into the cell and esterify these to coenzyme‐A for distribution into cellular lipids or energy generation via β‐oxidation. It was not known how the native lipid handling enzymes in yeasts, which normally process C16 and C18 saturated and monounsaturated fatty acids, would respond to CFA either generated intracellularly or supplied as an exogenous fatty acid in media. To investigate the response of native yeast lipid handling enzymes to the exotic fatty acid CFA, wild‐type *S. cerevisiae* BY4741, HBY14 engineered for increased lipid production and CBY14 strains expressing *EcCFA* (Table S2) were exogenously provided with CFA and fatty acid compositions of yeast lipids were determined and compared with yeast grown in standard media. CFA were incorporated into both TAG and PL in wild‐type BY4741 and lipid‐engineered HBY14 strains after feeding CFA (Figure 2A–D). CFA content in TAG was 2‐fold higher in HBY14 compared to the BY4741 strain but lower in PL (Figure 2B), attributed to the lipid accumulation engineering of HBY14 which promoted lipid flux towards TAG formation.

Exogenously supplied CFA also increased CFA content of PL and TAG in strain CBY14 by more than 2‐ and 3‐fold, respectively, above the strain without CFA supplement. Also, the content of CFA in PL of strain CBY14 given CFA exogenously reached 40% of PL (Figure 2C), significantly increased from 25% of strain CBY14 without supplementation. Taken together these results demonstrate that the native lipid metabolizing enzymes of *S. cerevisiae* accepts CFA as a substrate and inserts these into lipids within the cell. Furthermore, strain CBY14 which expresses CFA synthase has capacity to add further CFA in PL and TAG above the level resulting from gene expression alone in this strain.

Most yeast investigated to date do not produce CFA natively and therefore, CFA is an exotic fatty acid for them. In general, two major routes of TAG formation are present in yeast: the acyl‐CoA independent pathway and the acyl‐CoA dependent pathway.^[^ ^19^, ^20^, ^21^ ^]^ Exogenous fatty acids supplied to yeast may be activated into the coenzyme A form, then taken via the acyl‐CoA dependent route to synthesize TAG via acylation reactions with the glycerol backbone or alternatively to form PL.^[^ ^22^, ^23^ ^]^ CFA provided to *S. cerevisiae* strains BY4741 and HBY14 were identified in both PL and TAG fractions, suggesting the endogenous yeast enzymes such as acyl‐CoA synthetase can activate CFA, and acyl transferases can esterify CFA‐CoA to the glycerol backbone of TAG. Therefore, in the case of intracellular production of CFA, it is expected that CFA can be incorporated into TAG using the native CoA‐dependent pathway. However, the observation that only around 18% of TAG consist of CFA in the HBY14 strain supplemented with CFA suggests that the capacity of endogenous yeast enzymes to handle CFA is limited.

### 2.3 CFAs are generated in both *sn* positions of PLs in *EcCFA*‐expressing yeast especially where unsaturated fatty acids are enriched

In order to discern the site where EcCFA synthase acts on membrane lipids, particularly considering these lipids as the enzyme's primary substrates, an analysis of the fatty acid composition and their respective locations in PL was conducted in both wild type and engineered yeast strains. In the wild type BY4741 strain, the fatty acids C16:1 and C18:1 were found in both *sn*‐1 and *sn*‐2 positions with the majority in the *sn*‐2 position (Table 1). In strain CBY4741 expressing *EcCFA*, a noteworthy change was observed in the distribution of fatty acids within the lipids. Specifically, unsaturated fatty acids in both the *sn‐*1 and *sn‐*2 positions were substantially reduced, to the extent that they were absent in the *sn‐*1 position. Instead, these positions were predominantly occupied by CFA, with significantly higher quantities concentrated in the *sn‐*2 position. Clearly, the findings indicate that both C16:1 and C18:1 fatty acids located in the *sn‐*1 and *sn‐*2 positions of PL serve as suitable substrates for EcCFA synthase. Furthermore, as the *sn‐*2 position of *S. cerevisiae* lipids is naturally enriched with monounsaturated fatty acids, this enrichment contributes to the higher concentrations of CFA observed in the CBY4741 yeast strain.

**TABLE 1:** Fatty acid composition of *sn‐*1 and *sn‐*2 positions of yeast phospholipids (PL) in wild type and engineered yeast strains.

Strain	Position in PL	Percentage of fatty acid in PL (weight basis) ± SD				
		C16:1 cisΔ9	C16:0	C18:1 cis∆9	C18:0	CFA
BY4741 (wild type)	*sn*‐2	45.65 ± 5.38	7.01 ± 3.45	40.84 ± 0.03	5.78 ± 2.93	0.00
	*sn*‐1	8.48 ± 1.04	41.85 ± 2.42	17.78 ± 3.99	31.89 ± 2.61	0.00
CBY4741 (expressing *EcCFA*)	*sn‐*2	24.74 ± 2.47*	11.14 ± 2.39	24.05 ± 2.84*	8.17 ± 1.74	29.90 ± 1.68**
	*sn*‐1	0.00 **	41.56 ± 0.60	0.00 *	42.24 ± 2.02	16.20 ± 2.62*
CBY15 (expressing *EcCFA* ‐*ScOLE1*)	*sn*‐2	6.27 ± 5.54*	6.29 ± 3.56	10.91 ± 5.33*	5.31 ± 3.47	69.77 ± 5.88**
	*sn*‐1	0.00 **	39.73 ± 3.44	0.00 *	33.82 ± 1.61	26.45 ± 5.05*

*Note*: Values are means of triplicate experiments, error bars means standard deviation. “*”, “**”, and “***” denotes the effect of the factor was significant at *p* ≤ 0.05, *p* ≤ 0.01, and *p* ≤ 0.001, respectively. A two tailed Student's t‐test was used with BY4741 as the control strain.

Unsaturated fatty acid content of PL was increased by overexpression of Δ9 desaturase (*ScOLE1*), an enzyme that catalyzes the formation of a *cis‐*double bond between carbons 9 and 10 of palmitoyl (16:0) and stearoyl (18:0) CoA to form C16:1 and C18:1^8^ respectively. Strain CBY15 expressed both *ScOLE1* and *EcCFA*, and the CFA content in *sn*‐2 and *sn*‐1 positions more than doubled compared to strain CBY4741 expressing only *EcCFA* (Table 1). Concomitantly, the concentration of unsaturated fatty acids was heavily reduced in the *sn‐*1 and *sn‐*2 positions of PL in strain CBY15. Thus, the effect of co‐expression of *EcCFA* and *ScOLE1* on the composition of PLs demonstrates that CFA production in PL can be elevated by increased availability of Δ9 monounsaturated fatty acids in PL.

### 2.4 Metabolic engineering to improve substrate pool and enhance lipid accumulation increases CFA in TAG

**FIGURE 3:**
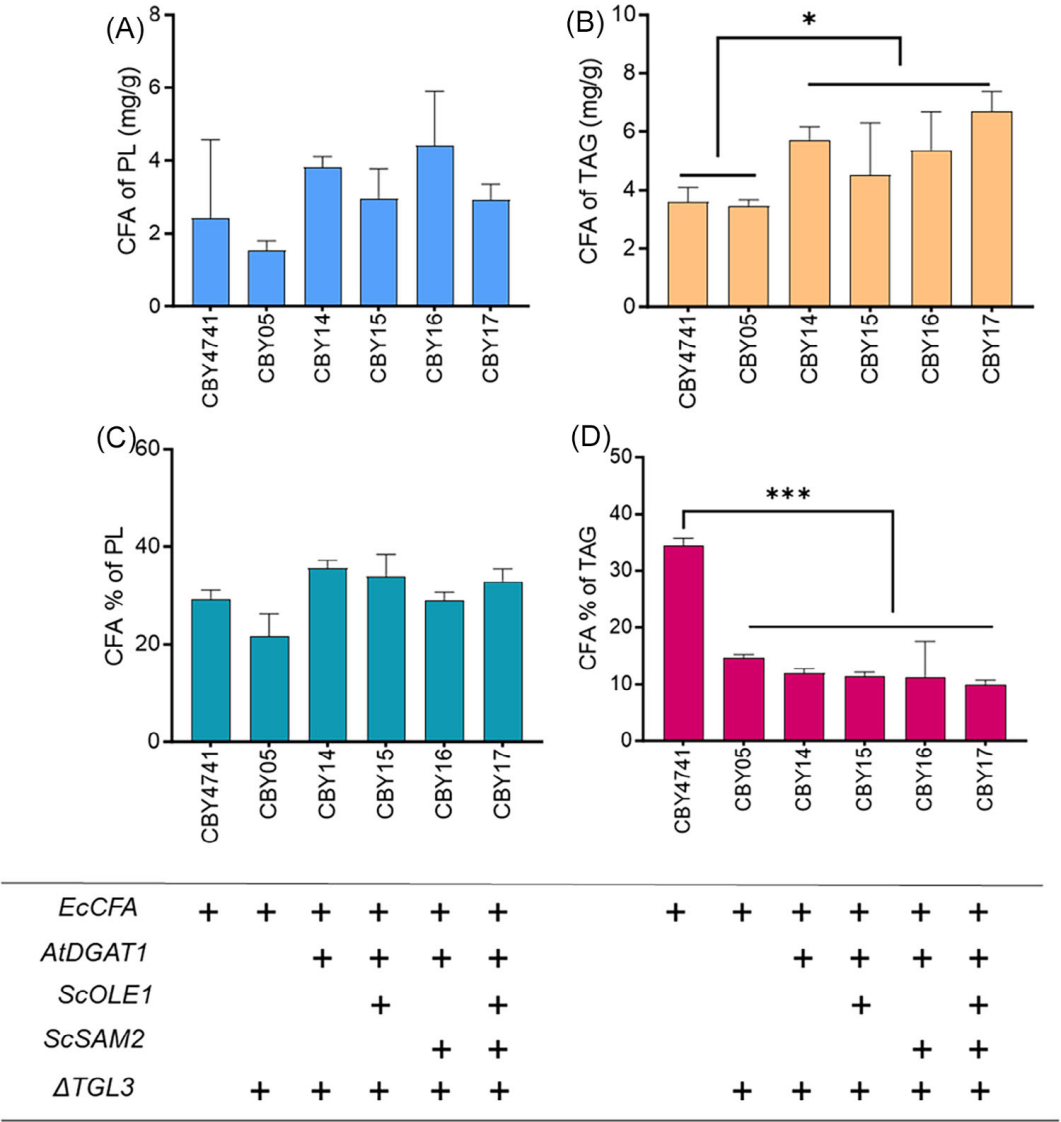
The effect of expression of *ScOLE1* and *ScSAM2* on CFA content and percentage in lipid fractions of engineered strains. (A) CFA in PL (mg g^−1^), (B) CFA in TAG (mg g^−1^), (C) percentage of CFA in PL fraction (%), (D) percentage of CFA in TAG fraction (%). “+”, genes expression. Values are means of triplicate experiments, error bars means standard deviation. “*”, “***” denotes the effect of the factor was significant at *p* ≤ 0.05 and *p* ≤ 0.001, respectively. A two tailed Student's t‐test was used.

Sequestering CFA in TAG and preventing TAG from being remobilized are strategies for stable storage of an exotic fatty acid within the cell. Previously, expression of diacylglycerol transferase 1 from *A. thaliana* (*AtDGAT1)* and deletion of *TGL3*, the major triacylglycerol lipase in *S. cerevisiae*, was demonstrated to effectively increase TAG levels in yeast.^[^ ^24^, ^25^ ^]^ Therefore, expression of *AtDGAT1* was added to the base strain expressing *EcCFA* and deletion in *TGL3* to produce strain CBY14, which showed increased CFA content in TAG above the base strain (Figure 3B) and significantly increased total TAG (Figure S2B). Furthermore, the Δ9 fatty acid desaturase, *ScOLE1*, was overexpressed in this strain (forming CBY15) to increase the pool of *EcCFA* substrate, however, its effect was marginally above that of CBY14 (Figure 3). As a separate investigation, the effect of increasing the pool of SAM, the co‐substrate of *EcCFA*, through overexpression of SAM synthase 2 (*ScSAM2*), was undertaken in the lipid engineered strain (forming CBY16). The impact of *ScSAM2* overexpression in this strain was also marginal in increasing CFA in TAG compared with CBY14 (Figure 3).

Interestingly, overexpression of both *ScOLE1* and *ScSAM2* in the lipid engineered strain (forming CBY17 strain) significantly increased CFA amount in TAG on a dried weight basis (Figure 3B) compared with CBY4741 that expressed only *EcCFA* synthase. The significant increase of CFA in CBY17 could be due to the effectiveness of increasing the substrate pool for *CFA* synthase and significantly increased lipid content, as compared to CBY4741 (Figure S2B). Intriguingly, CFA content as TAG in the CBY17 strain was among the highest in the engineered strains (Figure 3B) but among the lowest as a percentage of fatty acids in TAG (Figure 3D). Hence, increasing the lipid content through expression of *AtDAGT1* and Δ*TGL3* in this strain did not selectively increase CFA; standard fatty acids were also incorporated in TAG at a higher rate (Figure 3D). Identifying diacylglycerol acyltransferases that favor CFA as a substrate might be an effective approach to enhance CFA incorporation into TAG. Furthermore, CFA% of PL in CBY17 was similar to CBY4741 (Figure 3C) suggesting the formation of CFA on PL is no longer the rate‐limiting step for this strain but specific turnover of CFA from PL to TAG may be the new bottleneck for increasing CFA in TAG.

### 2.5 Increasing specific turnover of CFA from PL into TAG and preventing β‐oxidation

**FIGURE 4:**
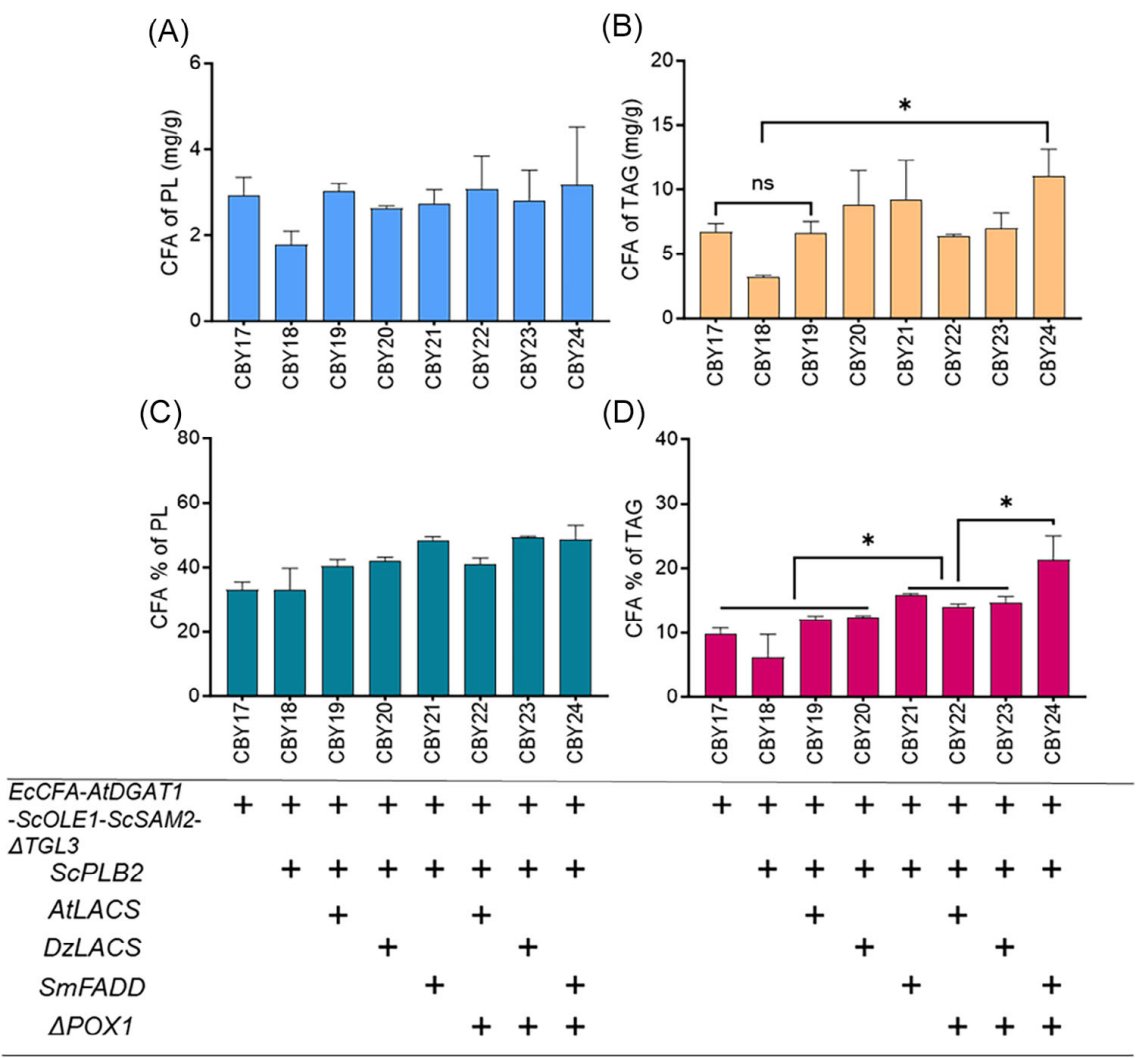
Impact of the engineering approaches to increase fatty acid flux from phospholipids into triacylglyercol by sequential expression of phospholipase, acyl‐CoA synthases and blocking of β‐oxidation on CFA content in TAG. (A) CFA in PL (mg g^−1^), (B) CFA in TAG (mg g^−1^), (C) percentage of CFA in PL fraction (%), and (D) percentage of CFA in TAG fraction (%). “+”, genes expression. Values are means of triplicate experiments, error bars means standard deviation. “*” denotes the effect of the factor was significant at *p* ≤ 0.05, ns, not significant. A two tailed Student's t‐test was used.

To increase the content of CFA in storage TAG, it was hypothesized that increased turnover of fatty acids in PL should lead to an increase in the cellular fatty acid pool and ultimately sequestration into TAG in lipid accumulating strains. Hence, a native phospholipase B2 (*ScPLB2)* which cleaves acyl chains at the *sn‐*1 and *sn‐*2 positions in the backbone of PL^[^ ^26^ ^]^ was selected for overexpression in the best performing strain to date (CBY17). The resulting strain expressing *ScPLB2* (CBY18) displayed some dramatic changes; while there was significantly reduced PL content compared to the parent strain (Figure S3A) and CFA levels were reduced in the PL fraction, CFA was also greatly reduced in TAG and was reflected in the low percentage of CFA to standard fatty acids in TAG (Figure 4A–D). This outcome indicated an enhanced release of CFA (and other fatty acids) from PL by *ScPLB2* expression but the fatty acids were not delivered to TAG as expected.

Phospholipases hydrolyze acyl chains and release free fatty acids, however, for ready incorporation into TAG fatty acid should be esterified with coenzyme A and β‐oxidation of fatty acids prevented, where possible. Therefore, to facilitate the conversion of free CFA released from PL into TAG, a range of acyl Coenzyme A (CoA) synthases were individually introduced into the engineered strain expressing *ScPLB2* (CBY18) and their impacts on CFA sequestration were compared. While the native acyl CoA synthases (ACS) of *S. cerevisiae* could activate CFA to CFA‐CoA, as shown by the CFA feeding study above, other candidates were investigated: *SmFADD* from *Sinorhizobium meliloti*, *AtLACS* from *A. thaliana* and *DuLACS* from *Durio zibethinus* as potentially having higher specificity for CFA over other fatty acids. *SmFADD* encodes an acyl‐CoA synthase in bacterial systems responsible for the activation of exogenous long‐chain fatty acids.^[^ ^27^, ^28^, ^29^ ^]^ *SmFADD* was proposed to be required for utilizing endogenous fatty acids released from membrane lipids for CFA synthesis in *S. meliloti*.^[^ ^30^ ^]^ In *D. zibethinus*, CFA are found in the seeds of this tropical fruit^[^ ^31^ ^]^ and its acyl‐CoA synthase has potential to selectively catalyze CoA formation with CFA. Seeds of *A. thaliana* were engineered to accumulate CFA^[^ ^32^ ^]^ suggesting *AtLACS* may be useful for CoA formation with CFA.

Among the three non‐native acyl‐CoA synthases, expression of *SmFADD* in yeast strain CBY18 to produce strain CBY21 was most useful in achieving an increase in CFA in TAG and CFA% (Figure 4B,D). *AtLACS* or *DuLACS* expression in the CBY18 strain, to form CBY19 and CBY20 respectively, had similar impacts on CFA content in PL and TAG but were less effective than *SmFADD* (Figure 4). Finally, the combined effect of expressing acyl‐CoA synthases and preventing β‐ oxidation of fatty acids was investigated by deleting the peroxisomally‐located acyl‐CoA oxidase, *POX1*, in the strains CBY19‐21 to generate CBY22‐24, respectively. The impact of *SmFADD* expression and *POX1* deletion (giving strain CBY24) resulted in the best outcome to date; CFA content in TAG was 3‐fold higher than CBY18 on a dried weight basis (Figure 4B) and the CFA proportion in TAG increased by >10% compared with CBY18 (Figure 4D). Therefore, the issues of the depletion of fatty acids and PL caused by expression of *SmPLB2* in CBY18 were effectively addressed by increasing fatty acid esterification to CoA and inhibiting of fatty acid β‐oxidation.

## 3 CONCLUSIONS

Elucidating the biosynthesis and storage mechanisms of unconventional or exotic fatty acids in yeasts is of critical importance for advancing yeast's application in precision fermentation of high‐value lipids. In this study, we employed a systematic approach to investigate the synthesis mechanism of CFA, a typical cycloalkane, in yeast, encompassing the subcellular localization of the cyclopropanation enzyme, *EcCFA* synthase, the response of native yeast lipid handling enzymes to the exotic fatty acid CFA, and the positional analysis of CFA in PL. Leveraging this information, we investigated potential limitations of precursors of CFA in PL, enhanced CFA turnover from PL to acyl‐CoA esters and increased the CFA storage in TAG by increasing the flux towards TAG synthesis.

While these strategies led to significant increases in CFA stored as TAG (up to ∼12 mg g^−1^ DCW) and >22% of fatty acids in TAG as CFA, significant new information on lipid engineering in yeast was also achieved. Specifically, it was revealed that *EcCFA* synthase when expressed in yeast, acts on monounsaturated fatty acids attached to membrane PL. Increasing the supply of monounsaturated fatty acid in PL resulted in increased CFA although this was concentrated in membranes. Improved flux of CFA from membranes to stable storage as TAG was achieved by overexpression of phospholipase and acyl‐CoA synthase whilst also preventing β‐oxidation of fatty acids. The systematic study approach employed here provides fundamental insights for the biosynthesis of exotic fatty acids in yeast, and serves as a valuable reference for the production of other exotic fatty acids that undergo similar conversion processes on phospholipid membranes in yeast, such as formation of long chain ω−3 polyunsaturated (PUFA), alkyne and hydroxylated fatty acids.

## 4 MATERIALS AND METHODS

### 4.1 Plasmid design and construction

*E. coli* Turbo Competent cells (NEB) were used for standard bacterial cloning and plasmid propagation. Selection and growth of *E. coli* was in Lysogeny Broth (LB) medium (VWR) at 37°C with aeration. Except generating competent cells, the LB medium was supplemented with appropriate antibiotics (ampicillin 100 μg mL^−1^, chloramphenicol 34 μg mL^−1^, or Kanamycin 50 μg mL^−1^).^[^ ^33^ ^]^

In this study, several genes were used from different organisms and their respective Accession Numbers were provided as follows: *EcCFA* (cyclopropane‐fatty‐acyl‐phospholipid synthase) from *E. coli* with Accession No.: NC_000913.3, *SmFADD* (fatty acyl‐CoA synthetase) from *Sinorhizobium meliloti* with Accession No.: NC_003047.1, *AtDGAT1* (diacylglycerol acyltransferase), and *AtLACS* (Long‐chain acyl‐CoA synthetase) from *A. thaliana* with Accession No.: NC_ 003071.7 and No.: NC_003071.7, respectively, *DuLACS* (Long‐chain acyl‐CoA synthetase) from *Durio zibethinus* with Accession No.: XP_022770776.1, *ScOLE1* (Δ9 Fatty acid desaturase), *ScPLB2* (lysophospholipase 2) and *ScSAM2* (S‐adenosylmethionine synthase 2) from *S. cerevisiae* with Accession No.: NC_001139.9, No.: NC_ 001145.3 and No.: NC_ 001136.10, respectively. All genes were codon‐optimised for *S. cerevisiae* and cloned into a pESC vector (Agilent, USA) under *GAL1* or *GAL10* promoters with different yeast auxotrophic markers (*URA3, HIS3, LEU2*). Each gene was designed with a Kozak sequence AAACA at the 5′ end to increase expression^[^ ^34^ ^]^ were ligated into BamHI‐XhoI, SpeI‐BglII, and BamHI‐XhoI, SpeI‐BglII, respectively, to generate the expression plasmids used in this study. Besides, the fusion construct *EcCFA*‐*GFP* was obtained by inserting *GFP* sequence into the pESC‐*EcCFA* plasmid at the N‐terminus of *EcCFA* under the *GAL1* promoter (Agilent, USA). A flexible linker GGGGSGGGGS was introduced between the peptide sequences of *GFP* and *EcCFA*. All plasmids in this study were listed in Table S1.

### 4.2 Yeast strain construction, validation and culture conditions

Model yeast *S. cerevisiae* BY4741 [ATCC 4040002] (MATa *his3Δ1 leu2Δ0 met15Δ0 ura3Δ0*) was used as the starting wild‐type strain in this study, which was maintained in yeast extract peptone dextrose (YPD) medium (10 g L^−1^ yeast extract, 20 g L^−1^ bacteriological peptone, and 20 g L^−1^ glucose). The details of yeast transformation and colony PCR were described in our previous study.^[^ ^35^ ^]^ Yeast transformation was performed by the lithium acetate (LiOAc) protocol,^[^ ^36^ ^]^ and yeast transformants were selected and grown in appropriate synthetic complete minimal medium (SC medium) based on the specific auxotrophic requirements of the transformants. The composition of the SC medium consisted of a 6.7 g L^−1^ yeast nitrogen base without amino acids, 20 g L^−1^ of glucose, and an appropriate yeast synthetic drop‐out medium supplement. In addition, the *EcCFA*‐*GFP* construct was introduced into our previously engineered yeast strain HBY14.^[^ ^25^ ^]^ This strain is derived from BY4741 and expresses *AtDGAT1* (diacylglycerol acyltransferase from *A. thaliana*) while also featuring the deletion of triglyceride lipase 3 (*TGL3*). To confirm the successful incorporation of gene expression or knockout, verification was conducted through yeast colony PCR using the Phire Plant Direct PCR Master Mix (F160L, Thermo Fisher).

Deletion strains were created by knocking out specific genes, including *TGL3* (triacylglycerol lipase 3), *POX1* (acyl‐coenzyme A oxidase), *ARE1* (sterol O‐acyltransferase 1), and *ARE2* (sterol O‐acyltransferase 2), utilizing the iterative marker‐less CRISPR‐Cas9 genome editing method as described in the MoClo Yeast Toolkit (YTK)^[^ ^37^ ^]^ and Shaw et al.^[^ ^33^ ^]^ The design of guide RNAs (gRNAs) for the target gene was carried out using the CRISPR tool within Benchling. Subsequently, the gRNA sequences were compiled and assembled into the gRNA expression vector via the Golden Gate gene assembly method. Meanwhile, donor DNA consisting of 500 bp homology arms, both upstream and downstream of the target region, was transformed into the yeast with the SpCas9 plasmid to assist the homology‐directed repair at the double‐strand break. All these deletion strains were verified by colony PCR and Sanger sequencing. The detailed information of primers, gRNAs, landing pads, donor DNA and knockout strains can be found in the list of plasmids (Table S1), strains (Table S2), oligos (Table S3), and full gene sequences (Table S4), respectively.

The yeast strains were initially pre‐cultured in the SC medium with glucose for 24 h. Subsequently, seed cultures were transferred into 250 mL Erlenmeyer flasks, each containing 50 mL SC medium with 2% w/v galactose and 1% w/v raffinose, starting with an initial OD600 nm of 0.4. These cultures were then incubated at 30°C, 250 rpm for 72 h before being harvested. The addition of galactose was used to induce heterologous gene expression. Following flask fermentation, cells were collected after centrifugation, then washed twice with distilled water. Subsequently, the wet biomass was frozen at −80°C, then lyophilised overnight. The resulting dry biomass was weighed and used for lipid analysis and subsequent lipid yield calculations.

In the CFA feeding experiment, *cis‐*9,10‐Methyleneoctadecanoic acid (Santa Cruz Biotechnology, Inc. USA) was first dissolved in ethanol at a concentration of 0.5 M. Then, it was added into the yeast culture medium at a final concentration of 100 μM. Additionally, 0.01% v/v tergitol (Sigma, USA) was included in the medium. Tergitol, being a non‐ionic surfactant, facilitated the dispersion of CFA in the medium. As a control, a medium containing the same concentration of tergitol and the equvialent volume of ethanol but without CFAs was employed.^[^ ^38^, ^39^ ^]^

### 4.3 Visualization of lipid droplet and mitochondria using confocal fluorescence microscope

In strain CBY28 expressing *EcCFA*‐*GFP*, lipid droplets were treated with the lipid stain BODIPY 558/568 C12 (Life Technologies Australia Pty Ltd). Yeast cells from a 24‐h induction culture were harvested, and their concentration was diluted to 0.5 OD_600nm_. Then, they were incubated in a shaker for 30 min with 0.5 μL BODIPY 558/568 C12 stock solution, which was prepared at a concentration of 1 μg mL^−1^. Following the staining process, yeast cells were washed twice with fresh medium.^[^ ^40^ ^]^ Mitochondria Deep Red (Life Technologies Australia Pty Ltd) was used to stain the mitochondria in strain CBY28. Yeast cells that were collected after a 24 h induction culture were centrifuged. The supernatant was discarded, and the cell pellet was suspended in HEPES buffer (0.1 M, pH 7.4). Mitochondria Deep Red stock solution (1 mM) was added to the yeast solution at a final concentration of 25 nM. After incubation for 30 min, the stained cells were washed twice with HEPES buffer. Subsequently, the stained yeast cells were imaged by a Leica Microsystems SP5 confocal microscope with HCX PL APO 63×/1.4 OIL CS oil‐immersion objective under the appropriate excitation lasers for the fluorophores. The images were analyzed by Leica LAS X (Leica Microsystems, Inc.) microscope control software.

### 4.4 Lipid analysis and quantification

Lipid extraction and separation using thin layer chromatography were referred to our previous study.^[^ ^14^, ^41^ ^]^ Fatty acid methyl ester (FAME) were quantified by gas chromatography (GC, Agilent 7890A) fitted with a Flame Ionisation Detector (FID) as previously described.^[^ ^41^ ^]^ The detailed procedures of the lipid analysis and the positional analysis of fatty acids in PL were provided in the supplementary methods.

## AUTHOR CONTRIBUTIONS

W.J., H.P., and V.H.: Conceptualization and experiment design. W.J.: Conducted the wet‐lab experiments, data analysis, visualization, and wrote the original draft. V.H. and H.P.: Manuscript revision and polish. L.H., R.L.A., and V.H.: Project administration and supervision, funding acquisition. All authors reviewed and approved the final manuscript.

## CONFLICT OF INTEREST STATEMENT

The authors declare no conflicts of interest.

## Supporting information

Supporting information

## Data Availability

Data available on request from the authors.
