# Supplementary material for: Exploring engineering strategies that enhance de novo production of exotic cyclopropane fatty acids in Saccharomyces cerevisiae
Source: Biotechnol J. 2024 Feb 25;19(2):2300694. doi: 10.1002/biot.202300694 (PMC11475713; doi:10.1002/biot.202300694)
Supplement: Supplementary file 1 — Supporting information [file BIOT-19-2300694-s001.docx]

Supplementary materials for

# Exploring engineering strategies that enhance *de novo* production of exotic cyclopropane fatty acids in *Saccharomyces cerevisiae*

Wei Jiang^1, 2, 3, #^, Huadong Peng^2, 3, #^, Lizhong He^1^, Rodrigo Lesma-Amaro^2, 3^, Victoria S. Haritos^1*^

^1^ Department of Chemical and Biological Engineering, Monash University, Clayton, Victoria, Australia

^2^ Imperial College Centre for Synthetic Biology, Imperial College London, London, UK

^3^ Department of Bioengineering, Imperial College London, London, UK

^#^ Wei Jiang and Huadong Peng share the co-first authorship.

*Correspondence to victoria.haritos@monash.edu

### **Methods of lipid analysis and quantification**

### **Lipid extraction and separation using thin layer chromatography**

Yeast cells after harvest were stored at -80℃ and freeze-dried under a vacuum overnight. Lipid extraction and separation using thin layer chromatography were referred to our previous study ^[1, 2]^. For the total fatty acids quantification, 20 μL 10 mg/mL tridecanoic acid was added to 20 ~ 30 mg dry yeast cells as the internal standard, then converted into fatty acid methyl esters (FAMEs) via the addition of 2 mL acidic methanol (methanol/hydrochloric acid/chloroform 10:1:1, v/v/v) and heated at 90°C for 1 h. Once cooled, the sample was washed with 0.9% NaCl solution and extracted with 2 mL hexane for the following gas chromatography (GC, Agilent 7890A) fitted with a Flame Ionisation Detector (GC-FID) analysis.

To analyse the CFA content in both TAG and polar lipid fractions, total lipids were extracted from 20 to 30 mg of dry yeast cells using a modified version of the Bligh and Dyer method ^[3]^, employing a mixture of methanol/chloroform (2:1, v/v). 0.2 mg of glyceryl tritridecanoate was incorporated as an internal standard during the extraction process. The mixture was vortexed for 6 min with glass beads, then 0.5 mL chloroform and 0.5 mL Millipore H_2_O were used to isolate lipids from the mixture. After centrifugation, 0.5 mL chloroform containing lipids was transferred into a clean 2 mL GC vial, then dried under nitrogen gas and redissolved in chloroform. Next, thin layer chromatography (TLC) silica gel plates (L × W 20 cm × 20 cm; Sigma-Aldrich) were used to separate lipids redissolved in chloroform. The lipid components were separated using hexane/diethyl ether/acetic acid (70:30:1, v/v/v) as the mobile phase. Then iodine vapour was used to visualise lipid spots. Phospholipids (PLs), TAGs, and other lipids were scraped from the corresponding region of the TLC plate, and individual samples were methylated by reacting them with 2 mL acidic methanol (methanol/hydrochloric acid/chloroform 10:1:1, v/v/v) and heated at 80°C for 1 h. The resulting fatty acid methyl esters were extracted into hexane and analysed by GC-FID as described below.

### **Positional analysis of fatty acids in phospholipids**

Phospholipids (PLs) were carefully scraped from the corresponding region of the above thin-layer chromatography (TLC) plate and subsequently dissolved in 0.5 ml of borate buffer (0.5 M, pH 7.5, containing 0.4 mM CaCl_2_). After sonication, 5 U of phospholipase A2 from honeybee venom (Sigma-Aldrich) was added to digest PLs plus diethyl ether (2 mL) and the mixture then vortexed for 2 h at 22°C. The ether phase was removed by evaporation, then 0.3 mL 1 M HCl was added to stop the digestion of PLs. The reaction mixture was extracted with chloroform/methanol (2:1, v/v), then dried under nitrogen stream and redissolved in chloroform. Next, the extract was separated by TLC in chloroform/methanol/ammonia/water (70:30:4:2, v/v/v/v) and lipid spots visualised using iodine vapour. Spots corresponding to released free fatty acids and lysophospholipids were located, then carefully scraped from the plates and methylated directly as described above. Finally, the fatty acid methyl esters were measured by GC-FID as described below.

### **Fatty acid methyl esters quantification by gas chromatography**

FAME were quantified by gas chromatography (GC, Agilent 7890A) fitted with a Flame Ionisation Detector (FID) as previously described ^[1]^. GC conditions included helium as the carrier gas, and separation was achieved using a DB-Fast FAME capillary column (Agilent J&W 30 m ×0.25 mm ×0.25 μm). Injections were made in split mode (10:1), the initial column temperature was 80°C for 0.5 min, then increased at 20°C/min to 175°C, then at 10°C/min to 185°C held for 0.5 min, then heated at 7°C/ min to 230°C and held for 10 min. The injection port and flame ionisation detector were 250°C. Fatty acids in yeast were identified by retention time in comparison with the retention times of 37 individual fatty acids in the mixed fatty acid standard (Supelco 37 Component, Sigma Aldrich). Each fatty acid was baseline-separated in the GC-FID program and individual calibration curves were generated for each component over the range of 1- 40 μg/mL. CFAs (as FAME) was analysed separately using a pure standard of cis-9,10-methylene octadecanoic acid (ab144075, Abcam, Inc.) and a calibration curve was generated by analysis of this standard diluted in hexane (1- 40 μg/mL). C17CFA and C19CFA as FAME eluted at 8.43 min and 10.43 min, respectively, and were clearly separated from all other 37 fatty acids and expressed as total CFA.

Table S1 Plasmids used in this study

| Plasmid | Describtion | Reference |
| --- | --- | --- |
| WJP1 | *pYES-URA3-pGAL1-EcCFA* | ^[4]^ |
| WJP2 | *pESC-URA3-pGAL1-EcCFA GFP* | This study |
| WJP3 | *pESC-LEU2-pGAL1-AtDGAT1* | ^[4]^ |
| WJP4 | *pESC-LEU2-pGAL1-AtDGAT1-pGAL10-SmfadD* | This study |
| WJP5 | *pESC-LEU2-pGAL1-AtDGAT1-pGAL10-AtLACS* | This study |
| WJP6 | *pESC-LEU2-pGAL1-AtDGAT1-pGAL10-DzLACS* | This study |
| WJP7 | *pESC-URA3-pGAL1-EcCFA-pGAL10-PLB2* | This study |
| WJP8 | *pESC-LEU2-pGAL1-AtDGAT1-PGAL10-ScOLE1* | This study |
| WJP9 | *pESC-LEU2-pGAL1-AtDGAT1-PGAL10-ScSAM2* | This study |
| WJP10 | *pESC-HIS3-pGAL1-ScSAM2-pGAL10-ScOLE1* | This study |
| pYTK001 | Entry vector | ^[5]^ |
| pWS2069 | SpCas9 gRNA gap repair vector | ^[6]^ |
| pHP129 | *POX1* gRNA1 | This study |
| pHP130 | *POX1* gRNA2 | This study |
| pHP131 | *ARE1* gRNA1 | This study |
| pHP132 | *ARE1* gRNA2 | This study |
| pHP133 | *ARE2* gRNA1 | This study |
| pHP134 | *ARE2* gRNA2 | This study |
| pHP141 | *POX1* donor DNA | This study |
| pHP142 | *ARE1* donor DNA | This study |
| pHP143 | *ARE2*  donor DNA | This study |

Table S2 Strains used in this study

| **Strains** | **Genotype** | **Parental strains** | **Reference** |
| --- | --- | --- | --- |
| BY4741 | Parental strain, MATa his3Δ1 leu2Δ0 met15Δ0 ura3Δ0 | - | Lab stock |
| HBY05 | BY4741*ΔTGL3* | BY4741 | ^[7]^ |
| sHP122 | BY4741*ΔPOX1* | BY4741 | This study |
| sHP123 | BY4741*ΔTGL3ΔPOX1* | BY4741*ΔTGL3* | This study |
| sHP124 | BY4741*ΔTGL3ΔARE1ΔARE2* | BY4741*ΔTGL3* | This study |
| sHP125 | BY4741*ΔTGL3ΔPOX1ΔARE1ΔARE2* | BY4741*ΔTGL3* | This study |
| HBY14 | BY4741*AtDGAT1-ΔTGL3* | *ΔTGL3* | *^[7]^* |
| CBY4741 | BY4741*EcCFA* | BY4741 | ^[4]^ |
| CBY05 | BY4741*EcCFA-ΔTGL3* | HBY05 | ^[4]^ |
| CBY14 | BY4741*EcCFA-AtDGAT1-ΔTGL3* | CBY05 | ^[4]^ |
| CBY15 | BY4741*EcCFA -AtDGAT1-ScOLE1-ΔTGL3* | CBY05 | This study |
| CBY16 | BY4741*EcCFA-AtDGAT1-ScSAM2-ΔTGL3* | CBY05 | This study |
| CBY17 | BY4741*EcCFA-AtDGAT1-ScOLE1-ScSAM2-ΔTGL3* | CBY14 | This study |
| CBY18 | BY4741*EcCFA-AtDGAT1-ScOLE1-ScSAM2-ScPLB2-ΔTGL3* | CBY16 | This study |
| CBY19 | BY4741*EcCFA-AtDGAT1-ScOLE1-ScSAM2-ScPLB2-AtLACS-ΔTGL3* | CBY16 | This study |
| CBY20 | BY4741*EcCFA-AtDGAT1-ScOLE1-ScSAM2-ScPLB2-DuLACS-ΔTGL3* | CBY16 | This study |
| CBY21 | BY4741*EcCFA-AtDGAT1-ScOLE1-ScSAM2-ScPLB2-SmFADD-ΔTGL3* | CBY16 | This study |
| CBY22 | BY4741*EcCFA-AtDGAT1-ScOLE1-ScSAM2-ScPLB2-AtLACS-ΔTGL3-ΔPOX1* | sHP123 | This study |
| CBY23 | BY4741*EcCFA-AtDGAT1-ScOLE1-ScSAM2-ScPLB2-DuLACS-ΔTGL3-ΔPOX1* | sHP123 | This study |
| CBY24 | BY4741*EcCFA-AtDGAT1-ScOLE1-ScSAM2-ScPLB2-SmFADD-ΔTGL3-ΔPOX1* | sHP123 | This study |
| CBY25 | BY4741*EcCFA-AtDGAT1-ScOLE1-ScSAM2-ScPLB2-AtLACS-ΔTGL3-ΔARE1-ΔARE2* | sHP124 | This study |
| CBY26 | BY4741*EcCFA-AtDGAT1-ScOLE1-ScSAM2-ScPLB2-DuLACS-ΔTGL3-ΔARE1-ΔARE2* | sHP124 | This study |
| CBY27 | BY4741*EcCFA-AtDGAT1-ScOLE1-ScSAM2-ScPLB2-SmFADD-ΔTGL3-ΔARE1-ΔARE2* | sHP124 | This study |
| CBY28 | BY4741*EcCFA-GFP-AtDGAT1-ΔTGL3* | HBY05 | This study |

**Table S3** Primers used in this study

| **Primer No. and Description** | **Sequences 5'-3'** | **Notes** |
| --- | --- | --- |
| oWJ001 CFA forward | CTTGTATTGAAGAAGTTTCTGTTCCAGATGATAATTG | yeast colony PCR for verfication of *EcCFA* and *EcCFA-GFP* gene |
| oWJ002 CFA reverse | CATCTTGCTACTCTCAAACCATTTTCTACAC |  |
| oWJ003 ScOLE1 forward | CCTACATCAGGTACAACAATAGAATTAATAGACG | yeast colony PCR for verfication of *ScOLE1* |
| oWJ004 ScOLE1 reverse | GAAGAATTTACCAGTTTCGTAGATTTCACCTC |  |
| oWJ005 ScSAM2 forward | GATCCAAACAATGTCTAAGTCAAAG | yeast colony PCR for verfication of *ScSAM2* |
| oWJ006 ScSAM2 reverse | GAGTTATTAAAATTCCAATTTCTTTGG |  |
| oWJ007 AtDGAT1 forward | GGATCCAAACAATGGCTATTTTAGATTC | yeast colony PCR for verfication of *AtDGAT1* |
| oWJ008 AtDGAT1 reverse | CTCGAGTTATGACATAGAACCCTTTC |  |
| oWJ009 SmfadD forward | GTAAACAATGGCCGAAGCCTCAACTC | yeast colony PCR for verfication of *SmfadD* |
| oWJ0010 SmfadD reverse | GATCTTCAACCTCTCAAGTCCTTTCTC |  |
| oHP005 URA3 5’ forward | GGGCGGATTACTACCGTT | Verification primers for the integration of URA3, LEU2 and HIS3 marker |
| oHP006 URA3 5’ reverse | GTAATGTTATCCATGTGGGC |  |
| oHP007 URA3 3’ forward | AGAGCACTTGAATCCACTGC |  |
| oHP008 URA3 3’ reverse | GATTTGGTTAGATTAGATATGGTTTC |  |
| oHP009 LEU2 5’ forward | CATAAATACCTTTCAAGC |  |
| oHP010 LEU2 5’ reverse | TACAATCCTTGCCCGTGATG |  |
| oHP011 LEU2 3’ forward | ACTCGTATCGCATGTCGGTG |  |
| oHP012 LEU2 3’ reverse | CTTCTTATGTTTTACATG |  |
| oHP013 HO 5’ forward | CACATCATTTTCGTGGATCC |  |
| oHP014 HO 5’ reverse | ACAGCGATGGAACTTACGGC |  |
| oHP015 HO 3’ forward | TATCGTGTTGCATCTGCGGC |  |
| oHP016 HO 3’ reverse | CTTTGGACTTAAAATGGCGT |  |
| oHP110 gRNAseq | CCCAGATGTTTTATACGGCGAGTC | universial primer for gRNA sequencing |
| oHP195 POX1 (YGL205W) Assembly 1 forward | AGATTTTTAGAGTCATCCCCGGAG | gRNA plasmids for POX1 |
| oHP196 POX1 (YGL205W) Assembly 1 reverse | AAACCTCCGGGGATGACTCTAAAA |  |
| oHP197 POX1 (YGL205W) Assembly 2 forward | AGATATGCTTGTCAATAAAATACA |  |
| oHP198 POX1 (YGL205W) Assembly 2 reverse | AAACTGTATTTTATTGACAAGCAT |  |
| oHP199 ARE1 (YCR048W) Assembly 1 forward | agatCTTCTGCGGAATTGAGTCTG | gRNA plasmids for ARE1 |
| oHP200 ARE1 (YCR048W) Assembly 1 reverse | aaacCAGACTCAATTCCGCAGAAG |  |
| oHP201 ARE1 (YCR048W) Assembly 2 forward | agatCGCTTTATTGAATTGCGTGG |  |
| oHP202 ARE1 (YCR048W) Assembly 2 reverse | aaacCCACGCAATTCAATAAAGCG |  |
| oHP203 ARE2 (YNR019W) Assembly 1 forward | agatCATTGCTAATTCATGAACGA | gRNA plasmids for ARE2 |
| oHP204 ARE2 (YNR019W) Assembly 1 reverse | aaacTCGTTCATGAATTAGCAATG |  |
| oHP205 ARE2 (YNR019W) Assembly 2 forward | agatATCTATAACAGTGGACGACG |  |
| oHP206 ARE2 (YNR019W) Assembly 2 reverse | aaacCGTCGTCCACTGTTATAGAT |  |
| oHP207 POX1 dDNA forward | CTTTTCTTAATTCTCTTTGTATTTATTCCTAGCGACG | POX1 donor DNA preparation |
| oHP208 POX1 dDNA reverse | GATTGTTACCATAGCAACTCATGTC |  |
| oHP209 POX1 dDNA forward 1 | TGTGCACGTCTCGTCGGGCGGCCGCCTTTTCTTAATTCTCTTTGTATTTATTCCTAGCGACG |  |
| oHP210 POX1 dDNA reverse 1 | TGTGCACGTCTCGCAGGATAACAGCATCGCAATACTAATTTATTATATTTTCTTTTTTTCTGTGATACTTATTAGTGTCAAGTGTG |  |
| oHP211 POX1 (YGL205W) forward (2) | TGTGCACGTCTCGCCTGCATCGGAAGTAGAGGTTTCCTGTTTTC |  |
| oHP212 POX1 (YGL205W) reverse (2) | TGTGCACGTCTCGGGTCGCGGCCGCGATTGTTACCATAGCAACT |  |
| oHP213 ARE1 donor DNA forward | CGCCCTCAGCGTCATCTT | ARE1 donor DNA preparation |
| oHP214 ARE1 donor DNA reverse | aaaaaGTATTACCCGAAACTTAAACAATGAATGG |  |
| oHP215 ARE1 (YCR048W) forward (1) | CACAAGCGTCTCCtcggGCGGCCGCCGCCCTCAGCGTCATCTT |  |
| oHP216 ARE1 (YCR048W) reverse (1) | CACAAGCGTCTCCTACAATAATATTCTTGCAATCTGTTTTGGCGCTCTTGCTGCAAGCCGTGCTGA |  |
| oHP217 ARE1 (YCR048W) forward (2) | CACAAGCGTCTCGTGTACACCTACCGTCACAAATTTTTACTAAGACACGTGTACCA |  |
| oHP218 ARE1 donor DNA reverse (2) | CACAAGCGTCTCGGGTCGCGGCCGCAAAAAGTATTACCCGAAACTTAAACAATGAATGG |  |
| oHP219 ARE2 donor DNA forward | CGCACTGAGGGTCGCCGAAGAGGAAGAGAGA | ARE2 donor DNA preparation |
| oHP220 ARE2 Donor DNA reverse | GATGATCTAAAATGGCAAATAGATTG |  |
| oHP221 ARE2 donor DNA forward (1) | GTATGGCGTCTCGTCGGGCGGCCGCCGCACTGAGGGTCGCCGAAGAGGAAGAGAGA |  |
| oHP222 ARE2 (YNR019W) reverse (1) | GTATGGCGTCTCGTCCACCTGATGCGGTTGTGTTTGTTATTGTTGC |  |
| oHP223 ARE2 (YNR019W) forward (2) | GTATGGCGTCTCCTGGACTAGCATGTATTTTCTGGCTCGGTATCTGC |  |
| oHP224 ARE2 (YNR019W) reverse (2) | GTATGGCGTCTCCggtcGCGGCCGCGATGATCTAAAATGGCAAATAGATTGG |  |
| oHP251 POX1 LP reverse | TTCCGATGCAGGATAACAGC | Landing pad barcode for POX1 |
| oHP252 ARE1 LP reverse | CGGTAGGTGTACAATAATAT | Landing pad barcode for ARE1 |
| oHP253 ARE2 LP reverse | CATGCTAGTCCACCTGATGC | Landing pad barcode for ARE2 |
| oHP265 POX1 Verify forward | CTTTCTCTCTCAACTCTGATCC | Verification primer POX1 |
| oHP266 ARE1 Verify forward | CGATATATCCAAACCACACC | Verification primer ARE1 |
| oHP267 ARE2 Verify forward | CGCTCAATTCATTACCGTC | Verification primer ARE2 |

**Table S4** Gene sequences used in this study

| Gene name | Microorganism | Sequencing (5'-3') codon-optimised for *S. cerevisiae* |
| --- | --- | --- |
| *EcCFA* | *E coli* | ATGTCATCTTCTTGTATTGAAGAAGTTTCTGTTCCAGATGATAATTGGTACAGAATTGCAAATGAATTATTGTCAAGAGCAGGTATTGCTATTAATGGTTCTGCACCAGCTGATATTAGAGTTAAAAATCCAGATTTCTTTAAAAGAGTTTTGCAAGAAGGTTCATTAGGTTTGGGTGAATCATACATGGATGGTTGGTGGGAATGTGATAGATTGGATATGTTTTTCTCTAAAGTTTTGAGAGCTGGTTTGGAAAATCAATTACCACATCATTTTAAAGATACTTTGAGAATTGCAGGTGCTAGATTGTTTAATTTGCAATCTAAAAAGAGAGCTTGGATTGTTGGTAAAGAACATTACGATTTGGGTAATGATTTGTTTTCAAGAATGTTAGATCCATTCATGCAATACTCATGTGCTTACTGGAAAGATGCTGATAATTTGGAATCAGCTCAACAAGCAAAATTAAAAATGATTTGTGAAAAATTGCAATTAAAACCAGGTATGAGAGTTTTGGATATTGGTTGTGGTTGGGGTGGTTTAGCTCATTACATGGCTTCTAATTATGATGTTTCTGTTGTTGGTGTTACTATTTCTGCTGAACAACAAAAGATGGCTCAAGAAAGATGTGAAGGTTTGGATGTTACAATTTTATTGCAAGATTACAGAGATTTGAATGATCAATTTGATAGAATTGTTTCTGTTGGTATGTTTGAACATGTTGGTCCAAAGAATTACGATACATACTTTGCTGTTGTTGATAGAAATTTGAAACCAGAAGGTATTTTCTTATTGCATACTATTGGTTCTAAAAAGACTGATTTGAATGTTGATCCATGGATTAATAAGTACATTTTTCCAAATGGTTGTTTGCCATCAGTTAGACAAATTGCTCAATCTTCAGAACCACATTTTGTTATGGAAGATTGGCATAATTTTGGTGCTGATTATGATACAACTTTGATGGCTTGGTACGAAAGATTTTTGGCAGCTTGGCCAGAAATTGCTGATAATTATTCAGAAAGATTCAAAAGAATGTTTACTTATTACTTGAATGCATGTGCTGGTGCTTTTAGAGCTAGAGATATTCAATTATGGCAAGTTGTCTTTAGTAGAGGTGTAGAAAATGGTTTGAGAGTAGCAAGATGA |
| *PLB2* | *Saccharomyces cerevisiae* | ATGCAATTGAGAAACATCTTGCAAGCATCTTCATTAATTTCAGGTTTGTCTTTAGCTGCTGATTCTTCTTCTACTACTGGTGACGGTTATGCTCCATCTATTATTCCATGTCCATCAGATGATACTTCTTTAGTTAGAAATGCTTCAGGTTTGTCTACTGCTGAAACAGATTGGTTGAAGAAAAGAGATGCTTACACTAAGGAAGCATTACATTCATTTTTGTCAAGAGCTACATCAAACTTCTCTGATACTTCATTGTTATCTACATTGTTTTCATCTAATTCATCTAATGTTCCAAAAATTGGTATTGCTTGTTCTGGTGGTGGTTATAGAGCTATGTTAGGTGGTGCAGGCATGATTGCTGCAATGGATAATAGAACTGATGGTGCTAATGAACATGGTTTGGGTGGTTTGTTACAATCATCTACATACTTATCAGGTTTGTCTGGTGGTAATTGGTTAACTGGTACATTGGCTTGGAACAACTGGACTTCTGTTCAAGAAATCGTTGATCACATGTCTGAATCAGATTCTATTTGGAACATCACAAAGTCTATCGTTAATCCAGGTGGTTCAAATTTGACTTACACAATCGAAAGATGGGAATCTATCGTTCAAGAAGTTCAGGCTAAGTCAGATGCAGGTTTTAATATTTCATTATCTGATTTGTGGGCAAGAGCTTTATCATACAATTTCTTTCCATCTTTGCCAGATGCTGGTTCAGCATTAACTTGGTCATCTTTGAGAGATGTTGATGTTTTTAAAAACGGTGAAATGCCATTGCCAATTACAGTTGCAGATGGTAGATACCCAGGTACTACTGTTATTAATTTGAACGCTACTTTGTTCGAGTTTACTCCATTCGAAATGGGTTCATGGGACCCATCTTTGAACGCTTTTACTGATGTTAAGTATTTGGGTACTAACGTTACAAACGGTAAACCAGTTAATAAGGATCAATGTGTTTCTGGTTACGATAATGCTGGTTTTGTTATTGCTACATCAGCTTCTTTGTTTAATGAATTTTCTTTGGAAGCATCAACTTCTACATACTACAAGATGATCAACTCTTTTGCTAATAAGTATGTTAATAATTTGTCACAAGATGATGATGATATTGCAATCTATGCTGCAAATCCTTTTAAAGATACTGAATTTGTTGATAGAAATTACACATCATCTATCGTTGATGCTGATGATTTGTTTTTAGTTGATGGTGGTGAAGATGGTCAAAATTTGCCATTAGTTCCATTGATTAAGAAAGAAAGAGATTTGGATGTTGTTTTCGCTTTGGATATTTCTGATAACACTGATGAATCATGGCCATCTGGTGTTTGTATGACTAACACTTATGAAAGACAATACTCTAAGCAGGGTAAAGGCATGGCATTTCCATACGTTCCAGATGTTAACACATTTTTGAATTTGGGTTTGACTAATAAGCCAACTTTCTTTGGTTGTGATGCTAAAAATTTGACTGATTTGGAATACATTCCACCATTAGTTGTTTACATCCCAAATACAAAACATTCTTTTAATGGTAACCAATCAACTTTGAAGATGAATTACAATGTTACAGAAAGATTGGGTATGATTAGAAATGGTTTTGAAGCTGCAACTATGGGTAACTTCACAGATGATTCTAATTTCTTGGGTTGTATCGGTTGTGCTATCATCAGAAGAAAGCAAGAATCTTTAAATGCAACTTTGCCACCAGAATGTACAAAGTGTTTCGCTGATTACTGTTGGAACGGTACTTTATCAACATCTGCTAATCCAGAATTGTCTGGTAATTCTACTTACCAATCTGGTGCAATTGCTTCAGCAATTTCTGAAGCTACTGATGGTATTCCAATTACAGCATTGTTAGGTTCATCTACATCTGGTAACACTACATCAAATTCTACTACATCAACTTCATCTAACGTTACATCAAATTCTAATTCATCTTCAAACACTACATTAAATTCTAATTCTTCATCTTCATCTATTTCATCTTCAACTGCAAGATCATCTTCTTCTACTGCTAATAAGGCAAATGCTGCTGCTATTTCTTATGCTAACACTAACACATTGATGTCATTGTTAGGTGCTATTACTGCATTATTTGGTTTGATTTGATAA |
| *AtDGAT1* | *Arabidopsis thaliana* | ATGGCTATTTTAGATTCAGCAGGTGTTACTACAGTTACTGAAAATGGTGGTGGTGAATTTGTTGATTTGGATAGATTGAGAAGAAGAAAGTCAAGATCAGATTCTTCAAACGGTTTGTTATTGTCTGGTTCAGATAACAACTCTCCATCAGATGATGTTGGTGCTCCAGCAGATGTTAGAGATAGAATCGATTCTGTTGTTAACGATGATGCTCAAGGTACAGCTAATTTGGCAGGTGACAATAATGGTGGTGGTGACAACAACGGTGGTGGTAGAGGTGGTGGTGAAGGTCGTGGTAATGCTGATGCAACTTTTACATATAGACCATCAGTTCCAGCTCATAGAAGAGCAAGAGAATCCCCATTGTCCTCAGATGCTATTTTTAAGCAATCACATGCAGGTTTGTTTAATTTGTGTGTTGTTGTTTTGATTGCTGTTAATTCAAGATTGATCATCGAAAATTTGATGAAGTACGGTTGGTTGATCAGAACTGATTTCTGGTTTTCTTCAAGATCATTAAGAGATTGGCCATTGTTTATGTGTTGTATTTCTTTATCAATCTTCCCATTGGCTGCTTTTACTGTTGAAAAATTAGTTTTGCAAAAGTACATCTCTGAACCAGTTGTTATTTTCTTGCATATCATCATCACTATGACAGAAGTTTTGTACCCAGTTTACGTTACTTTGAGATGTGATTCTGCATTTTTGTCAGGTGTTACTTTGATGTTGTTGACATGTATCGTTTGGTTGAAATTGGTTTCTTATGCACATACATCATACGATATTAGATCATTGGCTAATGCTGCTGATAAGGCAAACCCAGAAGTTTCTTACTACGTTTCTTTAAAATCATTGGCTTACTTCATGGTTGCACCAACTTTGTGTTATCAACCATCTTACCCAAGATCAGCTTGTATTAGAAAAGGTTGGGTTGCTAGACAATTTGCAAAATTGGTTATTTTTACAGGTTTTATGGGTTTTATTATCGAACAATACATCAACCCAATTGTTAGAAATTCAAAGCATCCATTGAAGGGTGACTTGTTGTACGCAATCGAAAGAGTTTTGAAATTGTCTGTTCCAAATTTGTACGTTTGGTTGTGTATGTTCTACTGTTTCTTTCATTTGTGGTTGAACATCTTGGCTGAATTATTGTGTTTCGGTGACAGAGAATTTTACAAGGATTGGTGGAACGCAAAGTCTGTTGGTGACTACTGGAGAATGTGGAATATGCCAGTTCATAAGTGGATGGTTAGACATATCTATTTCCCATGTTTAAGATCAAAGATCCCAAAAACTTTAGCTATCATCATCGCATTTTTGGTTTCTGCTGTTTTTCATGAATTATGTATCGCTGTTCCATGTAGATTGTTTAAATTGTGGGCATTTTTGGGTATCATGTTCCAAGTTCCATTAGTTTTTATTACTAATTACTTGCAAGAAAGATTCGGTTCTACAGTTGGTAACATGATTTTCTGGTTCATTTTCTGTATCTTCGGTCAACCAATGTGTGTTTTGTTGTACTACCATGATTTGATGAACAGAAAGGGTTCTATGTCATAACTCGAGTAAGCTTGGTACCGCGGCTAG |
| *SmFADD* | *Sinorhizobium meliloti* | ATGGCCGAAGCCTCAACTCAACAAGCTGGTTCCTCTACCGCAAAAATCTGGTTAGGTTCTTATCCTCCTGGTGTCCCTGCCGAAATAGGTCCATTGACTTATAGATCAATTGGTGAATTTTTCGATCATGCTGTTGCACAATACTCATGGAGACCAGCTTTTACATGTATGGGTAAAGCTTTAACTTTTTCTGATTTGAATACTCATTCTGCTAAAATTGGTGCTTGGTTGCAATCATTAGGTTTGGCTAAAGGTGACAGAGTTGCTGTTATGATGCCAAATATTTTACAAAATCCAGTTATTGTTTACGGTATTTTGAGAGCAGGTTTTACTGTTGTTAATGTTAATCCATTGTATACTCCAAGAGAATTGGAACATCAATTGGTTGATGCAGGTGCTAAAGCAATTTTTGTTTTGGAAAATTTTGCTCATACAGTTGAACAAGTTTTGGCTAGAACAGAAGTTAAACATGTTGTTGTTGCTTCAATGGGTGACATGTTGGGTGCTAAAGGTGCTATTGTTAATTTGGTTGTTAGAAGAGTTAAAAAGTTGGTTCCAGCTTGGTCAATTCCAGGTCATTTGTCTTTTAAAACTGTTTTGGCAAAAGGTGCTACTTTAGGTTTTAAAAGACCAAATGTTGCTCCAGGTGACGTTGCATTTTTGCAATACACAGGTGGTACTACAGGTGTTTCTAAAGGTGCAACTTTGACTCATGCAAATTTGTTATCAAATATGGCACAAATGGAATTATGGTTGAATACTGCATTTTTAAGAAAACCAAGACCAGAATCTTTGACTTTTATGTGTGCTTTGCCATTATACCATATTTTTGCTTTGACAGTTAATTCTTTGATGGGTTTAGCTACTGGTGGTAATAATATTTTGATTCCAAATCCAAGAGATATTCCAGCTTTTGTTAAAGAATTAGGTAGATATAGAACTAATATTTTTCCAGGTTTGAATACTTTGTTTAATGCTTTGATGAATAATTCTGAATTCAGAAAATTGGATTTTTCTTCATTAATTTTGACTTTTGGTGGTGGTATGGCTGTTCAAAGACCAGTTGCTGAAAGATGGTTGGAATTAACTGGTTGTCCAATTCATGAAGGTTATGGTTTGTCAGAAACTTCTCCAGTTGCAACTGCTAATAGATTGGATACTGATGATTTTACAGGTACTATTGGTATTCCATTGCCATCAACAGAAGTTGAAATTAGAGATGAAGATGGTAGAACTTTACCAGTTGGTGAAATTGGTGAAATTTGTATTAGAGGTCCACAAGTTATGGCTGGTTACTGGCAAAGACCAGAAGAAACAGCTAGAGCAATTTCACCAGATGGTTTCTTTAGAACAGGTGACGTTGGTTTTATGAATGCAGAAGGTTTGACAAAAATTGTTGATAGAAAGAAAGATATGATTTTGGTTTCAGGTTTTAATGTTTTTCCAAATGAAATTGAAGAAGTTGCAGCTACTCATCCAGGTATTTTGGAATGTGCTGCAATTGGTGTTGCTGATCCACATTCAGGTGAAGCTGTTAAATTGTTTGTTGTTAGAAAAGATCCAAATTTGACAGAAGAAGAAGTTAAAAGACATTGTGCAGCATCATTGACTAATTATAAAAGACCAAGATATGTTGAATTCAGAACTGAATTGCCAAAATCTAATGTAGGTAAAATCTTGAGAAAGGACTTGAGAGGTTGA |
| *ScOLE1* | *Saccharomyces cerevisiae* | ATGCCTACATCAGGTACAACAATAGAATTAATAGACGACCAATTTCCAAAGGACGACAGTGCCTCATCAGGTATAGTAGACGAAGTCGATTTGACTGAAGCTAATATTTTGGCTACTGGTTTGAATAAGAAAGCACCAAGAATTGTTAATGGTTTTGGTTCTTTGATGGGTTCTAAAGAAATGGTTTCTGTTGAATTTGATAAAAAGGGTAATGAAAAGAAATCTAATTTGGATAGATTATTGGAAAAAGATAATCAAGAAAAAGAAGAAGCTAAAACTAAAATTCATATTTCTGAACAACCATGGACTTTGAATAATTGGCATCAACATTTGAATTGGTTGAATATGGTTTTGGTTTGTGGTATGCCAATGATTGGTTGGTACTTTGCTTTGTCTGGTAAAGTTCCATTACATTTGAATGTTTTCTTGTTTTCAGTTTTCTATTACGCTGTTGGTGGTGTTTCTATTACTGCTGGTTACCATAGATTGTGGTCTCATAGATCATACTCTGCTCATTGGCCATTGAGATTATTTTATGCTATTTTTGGTTGTGCTTCTGTTGAAGGTTCTGCTAAATGGTGGGGTCATTCTCATAGAATTCATCATAGATACACTGATACATTGAGAGATCCATATGATGCTAGAAGAGGTTTGTGGTACTCTCATATGGGTTGGATGTTATTGAAACCAAATCCAAAATACAAAGCTAGAGCAGATATTACAGATATGACTGATGATTGGACAATTAGATTTCAACATAGACATTACATTTTGTTAATGTTGTTAACTGCATTTGTTATTCCAACATTGATTTGTGGTTACTTTTTCAATGATTACATGGGTGGTTTAATATACGCTGGTTTTATTAGAGTTTTTGTTATTCAACAAGCTACTTTTTGTATTAATTCTTTGGCACATTATATTGGTACTCAACCATTTGATGATAGAAGAACTCCAAGAGATAATTGGATTACTGCAATTGTTACTTTTGGTGAAGGTTACCATAATTTTCATCATGAATTTCCAACTGATTACAGAAATGCTATTAAATGGTATCAATACGATCCAACTAAAGTTATTATCTATTTGACTTCTTTGGTTGGTTTGGCTTACGATTTGAAAAAGTTTTCTCAAAATGCAATTGAAGAAGCTTTAATTCAACAAGAACAAAAGAAAATTAATAAGAAAAAGGCTAAAATTAATTGGGGTCCAGTTTTGACAGATTTGCCAATGTGGGATAAACAAACATTTTTGGCTAAATCTAAAGAAAATAAGGGTTTAGTTATTATTTCAGGTATTGTTCATGATGTTTCTGGTTACATTTCTGAACATCCAGGTGGTGAAACTTTGATTAAAACAGCATTGGGTAAAGATGCTACTAAAGCATTTTCTGGTGGTGTTTACAGACATTCAAATGCTGCACAAAATGTTTTGGCTGATATGAGAGTTGCTGTTATTAAAGAATCTAAAAATTCAGCAATCAGAATGGCATCCAAGAGAGGTGAAATCTACGAAACTGGTAAATTCTTCTAA |
| *ScSAM2* | *Saccharomyces cerevisiae* | ATGTCTAAGTCAAAGACATTTTTGTTTACTTCTGAATCAGTTGGTGAAGGTCATCCAGATAAAATTTGTGATCAAGTTTCAGATGCAATTTTGGATGCTTGTTTAGAACAAGATCCATTTTCTAAGGTTGCATGTGAAACTGCTGCAAAAACTGGTATGATCATGGTTTTCGGTGAAATCACTACAAAGGCTAGATTAGATTACCAACAAATCGTTAGAGATACTATTAAGAAAATTGGTTACGATGATTCTGCTAAGGGTTTCGATTACAAGACATGTAACGTTTTGGTTGCAATCGAACAACAATCACCAGATATTGCTCAAGGTTTGCATTACGAAAAATCTTTGGAAGATTTGGGTGCAGGTGACCAAGGTATTATGTTTGGTTACGCTACTGATGAAACACCAGAAGGTTTGCCATTGACTATCTTGTTGGCTCATAAATTGAATATGGCTATGGCAGATGCTAGAAGAGATGGTTCATTGCCTTGGTTAAGACCAGATACTAAGACACAAGTTACTGTTGAATACGAAGATGATAATGGTAGATGGGTTCCAAAGAGAATCGATACAGTTGTTATTTCAGCACAACATGCTGATGAAATTTCTACTGCTGATTTGAGAACACAATTACAAAAGGATATTGTTGAAAAAGTTATTCCAAAAGATATGTTGGATGAAAATACTAAATATTTTATTCAACCATCTGGTAGATTTGTTATTGGTGGTCCACAAGGTGACGCTGGTTTGACAGGTAGAAAGATCATCGTTGATGCATACGGTGGTGCTTCTTCAGTTGGTGGTGGTGCTTTTTCTGGTAAAGATTACTCAAAGGTTGATAGATCAGCTGCTTATGCTGCAAGATGGGTTGCTAAATCATTGGTTGCTGCTGGTTTGTGTAAGAGAGTTCAAGTTCAATTTTCTTATGCTATCGGTATTGCTGAACCATTGTCTTTACATGTTGATACATACGGTACTGCTACAAAGTCTGATGATGAAATCATCGAAATTATTAAGAAAAATTTCGATTTGAGACCAGGTGTTTTAGTTAAAGAATTGGATTTGGCAAGACCAATATATTTGCCAACTGCTTCATACGGTCATTTCACAAACCAAGAATACTCTTGGGAAAAGCCAAAGAAATTGGAATTTTAA |
| *EcCFA-GFP* |  | ATGTCAAAAGGTGAAGAATTGTTTACTGGTGTAGTTCCTATATTGGTCGAATTGGATGGTGACGTCAACGGTCATAAGTTTAGTGTCTCAGGTGAAGGTGAAGGTGACGCTACTTACGGTAAATTGACATTAAAATTCATTTGTACAACTGGTAAATTGCCAGTTCCATGGCCAACATTGGTTACAACTTTTGGTTACGGTGTTCAATGTTTTGCTAGATACCCAGATCATATGAAACAACATGATTTCTTTAAATCTGCAATGCCAGAAGGTTATGTTCAAGAAAGAACTATTTTCTTTAAAGATGATGGTAATTATAAAACTAGAGCTGAAGTTAAATTTGAAGGTGACACATTGGTTAATAGAATTGAATTGAAAGGTATTGATTTTAAAGAAGATGGTAATATTTTAGGTCATAAATTGGAATATAATTACAATTCTCATAATGTTTACATTATGGCTGATAAACAAAAGAATGGTATTAAAGTTAATTTTAAAATTAGACATAATATTGAAGATGGTTCTGTTCAATTAGCAGATCATTACCAACAAAATACACCAATTGGTGACGGTCCAGTTTTATTGCCAGATAATCATTACTTGTCTACTCAATCAGCTTTGTCTAAAGATCCAAATGAAAAGAGAGATCATATGGTTTTATTGGAATTTGTTACTGCTGCAGGTATTACACATGGTATGGATGAATTGTATAAAGGTGGTGGTGGTTCAGGTGGTGGTGGTTCTTCATCTTCTTGTATTGAAGAAGTTTCTGTTCCAGATGATAATTGGTACAGAATTGCAAATGAATTATTGTCAAGAGCAGGTATTGCTATTAATGGTTCTGCACCAGCTGATATTAGAGTTAAAAATCCAGATTTCTTTAAAAGAGTTTTGCAAGAAGGTTCATTAGGTTTGGGTGAATCATACATGGATGGTTGGTGGGAATGTGATAGATTGGATATGTTTTTCTCTAAAGTTTTGAGAGCTGGTTTGGAAAATCAATTACCACATCATTTTAAAGATACTTTGAGAATTGCAGGTGCTAGATTGTTTAATTTGCAATCTAAAAAGAGAGCTTGGATTGTTGGTAAAGAACATTACGATTTGGGTAATGATTTGTTTTCAAGAATGTTAGATCCATTCATGCAATACTCATGTGCTTACTGGAAAGATGCTGATAATTTGGAATCAGCTCAACAAGCAAAATTAAAAATGATTTGTGAAAAATTGCAATTAAAACCAGGTATGAGAGTTTTGGATATTGGTTGTGGTTGGGGTGGTTTAGCTCATTACATGGCTTCTAATTATGATGTTTCTGTTGTTGGTGTTACTATTTCTGCTGAACAACAAAAGATGGCTCAAGAAAGATGTGAAGGTTTGGATGTTACAATTTTATTGCAAGATTACAGAGATTTGAATGATCAATTTGATAGAATTGTTTCTGTTGGTATGTTTGAACATGTTGGTCCAAAGAATTACGATACATACTTTGCTGTTGTTGATAGAAATTTGAAACCAGAAGGTATTTTCTTATTGCATACTATTGGTTCTAAAAAGACTGATTTGAATGTTGATCCATGGATTAATAAGTACATTTTTCCAAATGGTTGTTTGCCATCAGTTAGACAAATTGCTCAATCTTCAGAACCACATTTTGTTATGGAAGATTGGCATAATTTTGGTGCTGATTATGATACAACTTTGATGGCTTGGTACGAAAGATTTTTGGCAGCTTGGCCAGAAATTGCTGATAATTATTCAGAAAGATTCAAAAGAATGTTTACTTATTACTTGAATGCATGTGCTGGTGCTTTTAGAGCTAGAGATATTCAATTATGGCAAGTTGTCTTTAGTAGAGGTGTAGAAAATGGTTTGAGAGTAGCAAGATGA |
| *DzLACS* | *Durio zibethinus* | ATGAAAGTTTTCTCTGCTAAAGTTGAAGATGGTAGAGAAGGTCAAGATGGTAAACCATCTATTGGTCCAGTTTACAGAAATTTGTTGGCTAAAAATGGTTACCCATCACCAGATCATGATATGTCTACTGCTTGGACATTATTTTCTTCATCTGTTCAAAAGCATCCAGGTAATAGAATGTTGGGTTGGAGAAATATTGTTGATGGTAAAGTTGGTCCATACATCTGGAAGACTTACAAGGAAGTTTACGATGAAATTTTACATATCGGTTCAGCTTTGAGAGCATCTGGTGCTGAACCAGGTTGTAGAGTTGGTATCTATGGTGCTAATTGTCCACAATGGATTATGGCAATGGAAGCTTGTGGTGCACATTCTTTAGTTTGTGTTCCATTGTACGATACATTAGGTCCAGGTGCAGTTAACTTCATCATCGATCATGCTGAAGTTGATTTCGTTTTCGTTCAAGATAAGAAAGTTAAAGAATTGTTAAATCCAAATTGTTCATCTGCACAAAGATTGAAGGCTATGGTTTGTTTCACTTCATTGACAGAAGAAGATAATGCTAAGGCATCACAAATGGGTATTAAAACTTACTCTTGGACAGAATTCTTGCATATGGGTAAAGAAAACCCACAAGAAATTTCTCCACCACAACCTTTTAATATCTGTACTATCATGTACACTTCAGGTACATCTGGTGACCCAAAAGGTGTTGTTTTGACTCATGAAACAATCGCTATCTTCGTTCATGGTATTGATTTGTTTTTGGATCAATTCGAAGATAAGATGACAGTTGATGATGTTTACTTATCATTTTTACCATTGGCTCATATCTTGGATAGAATGATCGAAGAATATTTCTTTCATAAGGGTGCATCTGTTGGTTACTACCATGGTAATTTGAAGGAATTGAGAGATGATATCATGGAATTGAAGCCAACATTTTTGGCTGGTGTTCCAAGAGTTTACGATATGATCCATGAAGGTATTAAGAAAGCATTGCAAGAATTGAGACCATTAAGAAGATGGATCTTCGATGCTTTGTACAACTACAAGTTGTCATGGATGAAGAGAGGTTACAAGCATAAGTACGCTTCTCCATTAGCAGATTTGTTGGCTTTTAGAAAGGTTAAGGCAAAGTTGGGTGGTAGAATCAGATTGTTGTTGTCAGGTGGTGCTCCATTATCATCTGAAGTTGAAGAATTCTTGAGAGTTACTTGTTGTGCATTTGTTGTTCAAGGTTATGGTTTAACTGAAACATGTGGTGCTTGTACAATTGGTTTTCCAGATGAAATGTGTATGGTTGGTGCAGTTGGTGCTCCAGCAGTTTACAACGAATTGAGATTGGAAGAAGTTTCTGATATGGGTTATAATCCATTGGGTAATCCACCATTTGGTGAAATTTGTGTTAGAGGTAAAACTATTTTCTCTGAATACTACAAGAATCCAGAATTAACTAGAGAATCTTTTAAAGATGGTTGGTTTCATACAGGTGACATTGGTCAAATGTTGCCAAACGGTGTTGTTAAGATCATCGATAGAAAGAAAAATTTGATTAAATTGTCTCAGGGTGAATACGTTGCTTTGGAATACTTAGAAAACGTTTACGGTTTTACACCAATTGTTGATGATGTTTGGGTTTACGGTAACTCTTTTAAATCTATGTTGGTTGCAGTTGTTGTTTTGCATGAAGAAAACGCTAAGAAATGGGCTAATTTGAATGGTTACACTGGTTCATTGTCTGAATTGTGTTCATTGTCTAAGTTGCAAAACTATGTTTTGTCAGAATTGAAGTCTACAGCTGAAAAGAATAAGATGAGAGGTTTCGAATTCATTAAGGGTGTTATTTTGGAACCAGTTCCATTTGATATGGAAAGAGATTTGGTTACTGCTACATTGAAAAAGAAAAGAAATAAGTTGTTGAAGTACTACCAAGCAGAAATCGATGAATTGTACCAAAATTTGGCTGCAAAGTTGTAA |
| *AtLACS* | *Arabidopsis thaliana* | ATGAAATCATTTGCTGCAAAAGTTGAAGAAGGTGTTAAAGGTATTGATGGTAAACCATCTGTTGGTCCAGTTTACAGAAATTTGTTGTCAGAAAAGGGTTTTCCACCAATTGATTCTGAAATTACTACAGCTTGGGATATTTTCTCTAAGTCAGTTGAAAAGTTCCCAGATAATAATATGTTGGGTTGGAGAAGAATCGTTGATGAAAAGGTTGGTCCATACATGTGGAAGACTTACAAGGAAGTTTACGAAGAAGTTTTGCAAATTGGTTCAGCATTAAGAGCTGCAGGTGCTGAACCAGGTTCTAGAGTTGGTATCTATGGTGTTAATTGTCCACAATGGATTATTGCTATGGAAGCATGTGCTGCACATACTTTGATCTGTGTTCCATTGTACGATACATTAGGTTCTGGTGCAGTTGATTACATCGTTGAACATGCTGAAATCGATTTCGTTTTCGTTCAAGATACAAAGATTAAAGGTTTGTTAGAACCAGATTGTAAGTGTGCAAAGAGATTGAAGGCTATCGTTTCTTTTACTAACGTTTCTGATGAATTGTCACATAAGGCATCTGAAATCGGTGTTAAGACATACTCTTGGATCGATTTCTTGCATATGGGTAGAGAAAAACCAGAAGATACTAATCCACCAAAGGCTTTTAATATCTGTACAATCATGTACACTTCTGGTACATCAGGTGACCCAAAAGGTGTTGTTTTGACTCATCAAGCTGTTGCAACATTCGTTGTTGGTATGGATTTGTACATGGATCAATTCGAAGATAAGATGACTCATGATGATGTTTACTTGTCATTTTTGCCATTGGCACATATCTTGGATAGAATGAACGAAGAATATTTCTTTAGAAAGGGTGCTTCTGTTGGTTACTACCATGGTAATTTGAACGTTTTGAGAGATGATATCCAAGAATTGAAGCCAACATACTTAGCAGGTGTTCCAAGAGTTTTCGAAAGAATTCATGAAGGTATTCAAAAGGCTTTGCAAGAATTGAACCCAAGAAGAAGATTCATTTTTAATGCATTGTACAAGCATAAGTTGGCTTGGTTGAATAGAGGTTACTCTCATTCAAAAGCTTCTCCAATGGCAGATTTCATCGCTTTTAGAAAGATCCGTGATAAGTTAGGTGGTAGAATTAGATTGTTAGTTTCAGGTGGTGCTCCATTGTCTCCAGAAATCGAAGAATTCTTGAGAGTTACTTGTTGTTGTTTTGTTGTTCAAGGTTATGGTTTGACTGAAACATTAGGTGGTACTGCATTGGGTTTTCCAGATGAAATGTGTATGTTAGGTACAGTTGGTATTCCAGCTGTTTACAACGAAATCAGATTGGAAGAAGTTTCAGAAATGGGTTACGATCCATTAGGTGAAAATCCAGCTGGTGAAATTTGTATCAGAGGTCAATGTATGTTCTCTGGTTACTACAAGAACCCAGAATTGACTGAAGAAGTTATGAAAGATGGTTGGTTTCATACAGGTGACATTGGTGAAATTTTGCCAAATGGTGTTTTGAAGATCATCGATAGAAAGAAAAATTTGATTAAATTGTCACAGGGTGAATACGTTGCTTTGGAACATTTGGAAAACATCTTCGGTCAAAACTCTGTTGTTCAAGATATCTGGGTTTACGGTGACTCTTTTAAATCAATGTTGGTTGCAGTTGTTGTTCCAAATCCAGAAACTGTTAATAGATGGGCTAAAGATTTGGGTTTTACAAAGCCATTCGAAGAATTGTGTTCATTTCCAGAATTAAAGGAACATATCATCTCAGAATTGAAGTCTACAGCAGAAAAGAATAAGTTGAGAAAGTTCGAATACATCAAGGCTGTTACTGTTGAAACAAAGCCATTCGATGTTGAAAGAGATTTGGTTACTGCAACATTGAAAAATAGAAGAAACAATTTGTTGAAGTACTACCAAGTTCAAATCGATGAAATGTACAGAAAGTTAGCTTCTAAGAAAATTTGA |

**A**

**B**


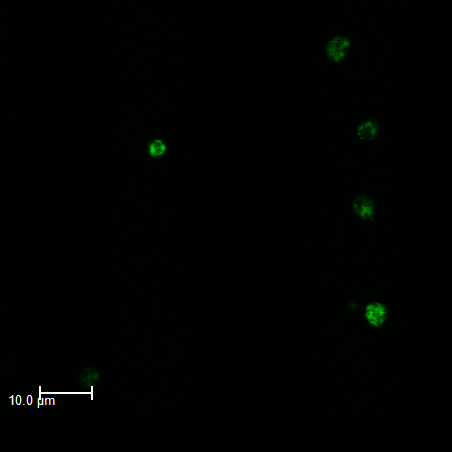

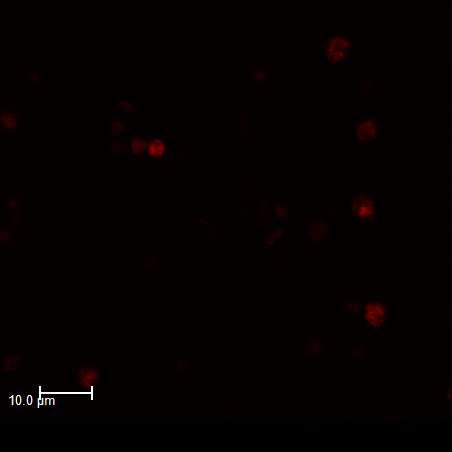

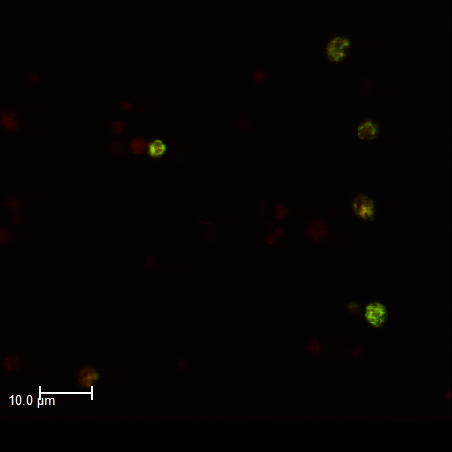

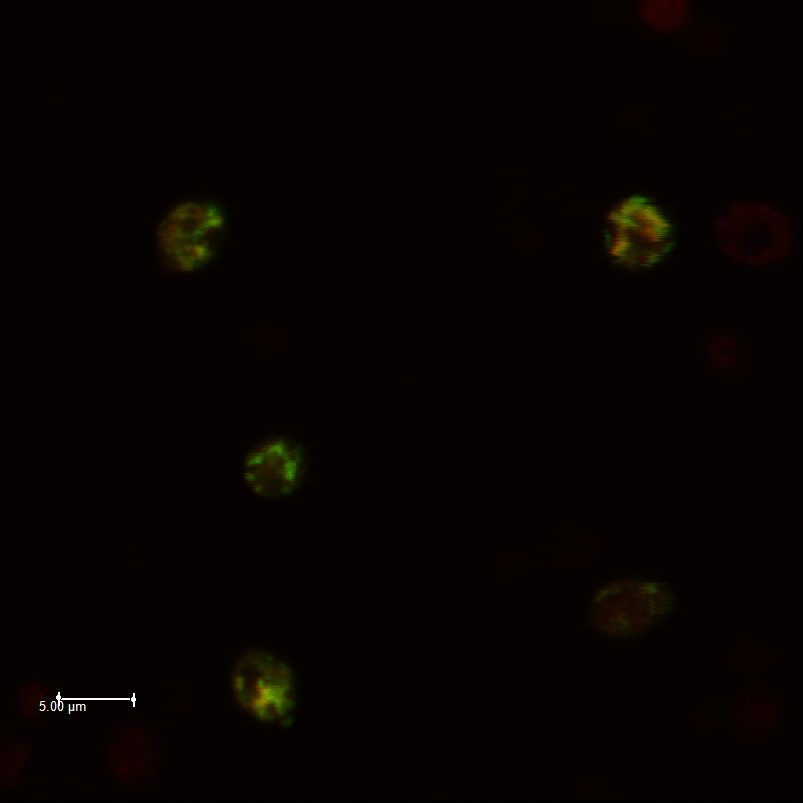


**B1**

**B2**

**B3**

**B4**


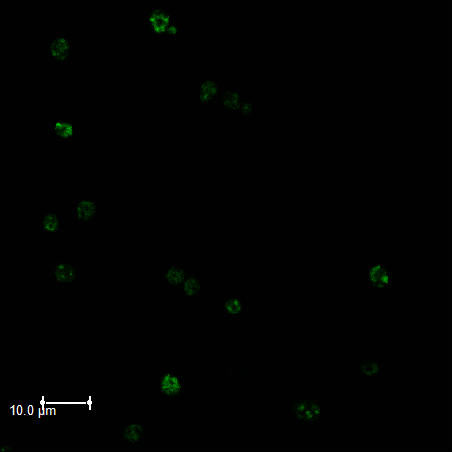

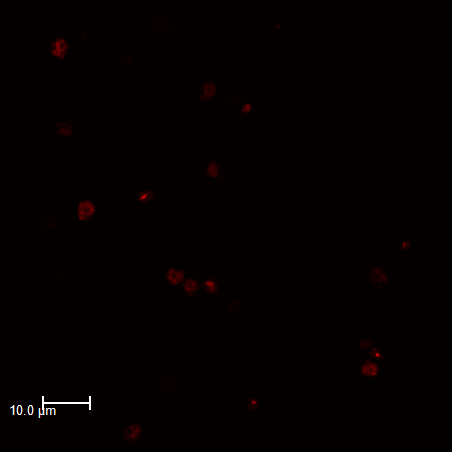


**C1**

**C2**


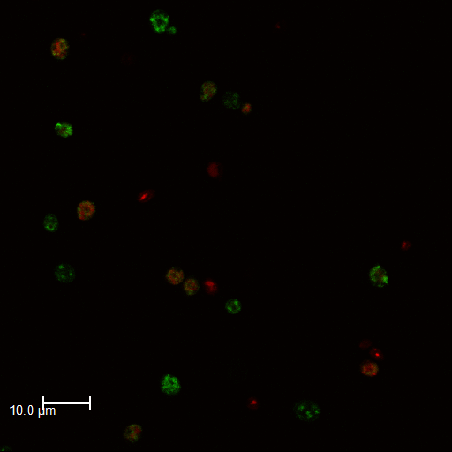

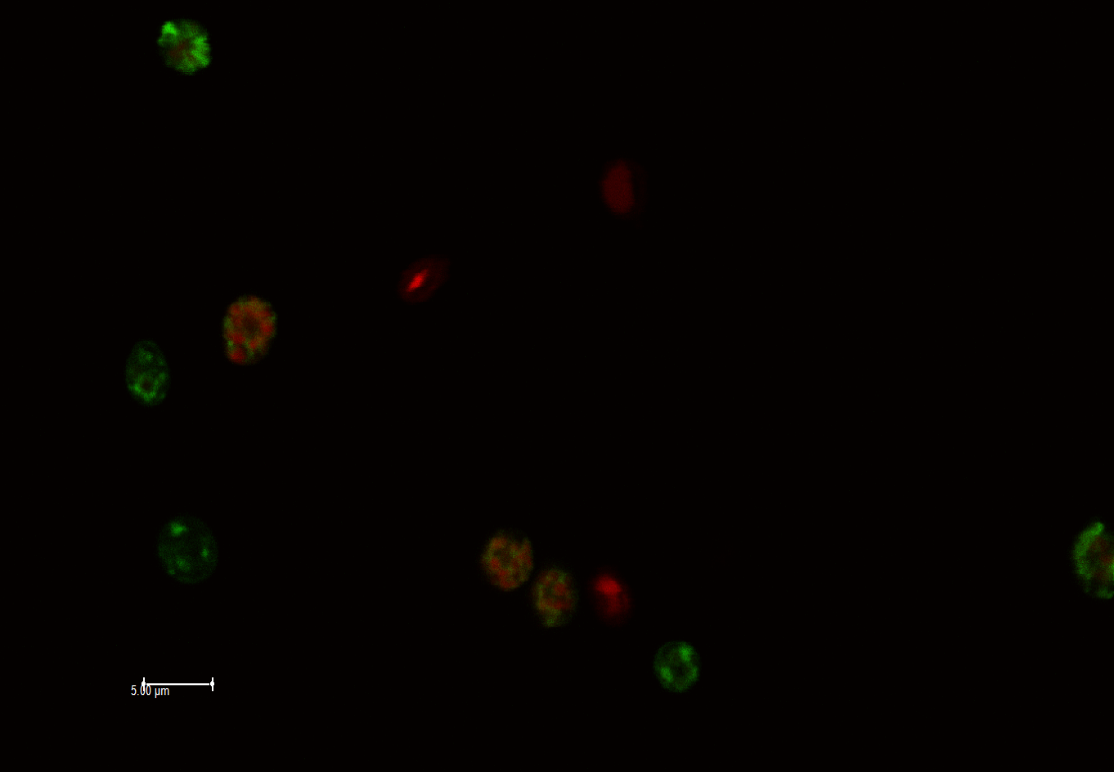


**C3**

**C4**

Figure S1 The expression and subcellular location of *Eccfa* when expressed in *S. cerevisiae* (A) Total CFA by weight % (DCW basis) and the percentage of CFA in total fatty acid (TFA) in strain CBY28 (*EcCFA-GFP-AtDGAT1-ΔTGL3*). Values are means of triplicate experiments, error bars means standard deviation. (B1), (C1) CBY28 strain yeast cells were imaged by confocal fluorescence microscopy, (B2),(C2) The mitochondria and lipid droplets of CBY28 strain yeast cells were stained by fluorescence dyes, Mitotracker deep red and Bodipy 558/568 C_12_, respectively, (B3), (B4) Overlay of *CFA*–*GFP* and Mitotracker deep red labelled mitochondria with different magnification, (C3), (C4) Overlay of *CFA*–*GFP* and of Bodipy 558/568 C_12_ labelled lipid droplets at a different magnification.


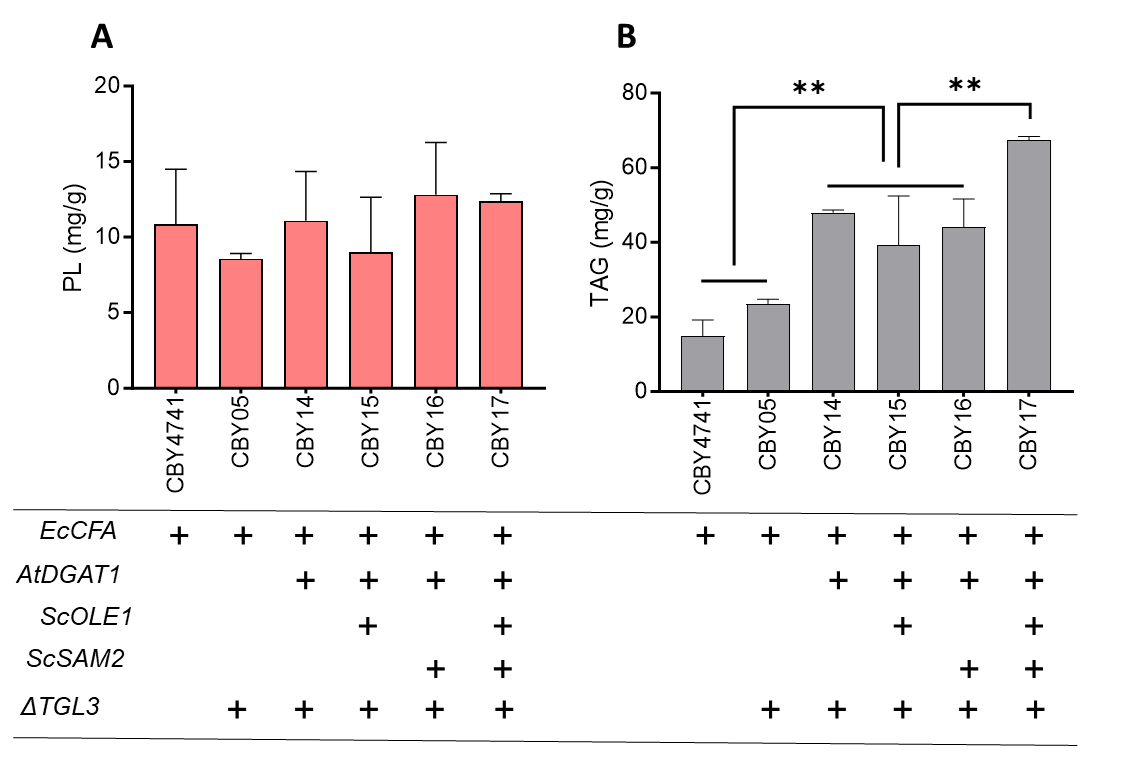


**Figure S2** The impact of *ScOLE1* and *ScSAM2* expression on (A) total phospholipid (PL) content (mg/g) and (B) total triacylglycerol (TAG) content (mg/g) in the engineered strains. Values are means of triplicate experiments, error bars means standard deviation. “*”, “**”, “***” denotes the effect of the factor was significant at p ≤ 0.05, p ≤ 0.01 and p ≤ 0.001, respectively. A two tailed Student’s t-test was used.


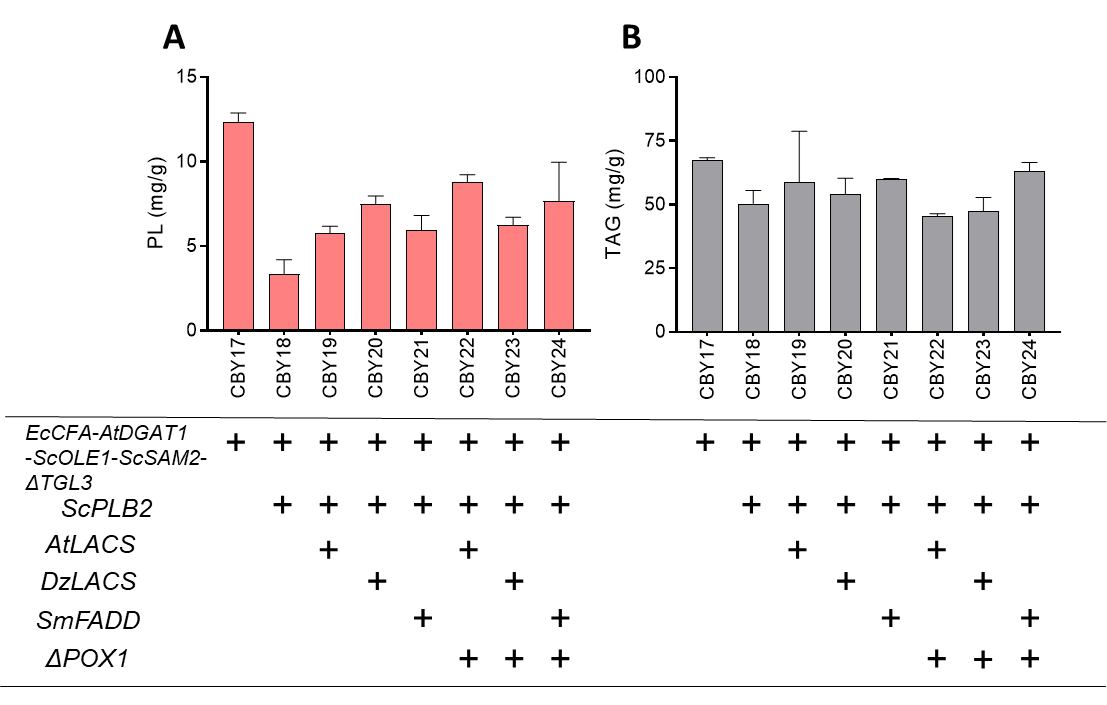


**Figure** **S3** The effect of *ScPLB2,* *SmFADD*, *AtLACS*, *DuLACS* expression, along with the knockout of *POX1* on (A) total phospholipid (PL) content (mg/g) and (B) total triacylglycerol (TAG) content (mg/g) in the engineered strains. Values are means of triplicate experiments, error bars means standard deviation.

**References**

[1] Peng, H., Moghaddam, L., Brinin, A., Williams, B., Mundree, S., Haritos, V. S., Functional assessment of plant and microalgal lipid pathway genes in yeast to enhance microbial industrial oil production. *Biotechnol Appl Biochem* 2018, *65*, 138-144.

[2] Peng, H., He, L., Haritos, V. S., Enhanced production of high-value cyclopropane fatty acid in yeast engineered for increased lipid synthesis and accumulation. *Biotechnol J* 2019, *14*, e1800487.

[3] Bligh, E. G., Dyer, W. J., A rapid method of total lipid extraction and purification. *Can J Biochem Physiol* 1959, *37*, 911-917.

[4] Peng, H., He, L., Haritos, V. S., Enhanced Production of High‐Value Cyclopropane Fatty Acid in Yeast Engineered for Increased Lipid Synthesis and Accumulation. *Biotechnology journal* 2019, *14*, 1800487.

[5] Lee, M. E., DeLoache, W. C., Cervantes, B., Dueber, J. E., A highly characterized yeast toolkit for modular, multipart assembly. *ACS synthetic biology* 2015, *4*, 975-986.

[6] Shaw, W. M., Yamauchi, H., Mead, J., Gowers, G.-O. F., Bell, D. J., Öling, D., Larsson, N., Wigglesworth, M., Ladds, G., Ellis, T., Engineering a model cell for rational tuning of GPCR signaling. *Cell* 2019, *177*, 782-796. e727.

[7] Peng, H., He, L., Haritos, V. S., Metabolic engineering of lipid pathways in Saccharomyces cerevisiae and staged bioprocess for enhanced lipid production and cellular physiology. *Journal of Industrial Microbiology and Biotechnology* 2018, *45*, 707-717.
